# Identification and characterization of microRNAs from in vitro*-*grown pear shoots infected with *Apple stem grooving virus* in response to high temperature using small RNA sequencing

**DOI:** 10.1186/s12864-015-2126-8

**Published:** 2015-11-16

**Authors:** Juan Liu, XueJiao Zhang, FangPeng Zhang, Ni Hong, GuoPing Wang, Aiming Wang, LiPing Wang

**Affiliations:** State Key Laboratory of Agricultural Microbiology, Wuhan, Hubei 430070 P. R. China; College of Plant Science and Technology, Huazhong Agricultural University, Wuhan, Hubei 430070 P. R. China; National Indoor Conservation Center of Virus-Free Germplasms of Fruit Crops, Wuhan, Hubei 430070 P. R. China; Lab of Key Lab of Plant Pathology of Hubei Province, Wuhan, Hubei 430070 P. R. China; Shihezi University, Shihezi City, Xinjiang Uyghur Autonomous Region 832003 P. R. China; Southern Crop Protection and Food Research Centre, Agriculture and Agri-Food Canada, London, N5V 4T3 ON Canada

**Keywords:** *Pyrus pyrifolia*, microRNA, High temperature, Small RNA sequencing, *Apple stem grooving virus*

## Abstract

**Background:**

MicroRNAs (miRNAs) have functions in diverse biological processes such as growth, signal transduction, disease resistance, and stress responses in plants. Thermotherapy is an effective approach for elimination of viruses from fruit trees. However, the role of miRNAs in this process remains elusive. Previously, we showed that high temperature treatment reduces the titers of *Apple stem grooving virus* (ASGV) from the tips of in vitro-grown *Pyrus pyrifolia* plants. In this study, we identified high temperature-altered pear miRNAs using the next generation sequencing technology, and futher molecularly characterized miRNA-mediated regulaton of target gene expression in the meristem tip and base tissues of in vitro-grown, ASGV-infected pear shoots under different temperatures.

**Results:**

Using in vitro-grown *P. pyrifolia* shoot meristem tips infected with ASGV, a total of 22,592,997 and 20,411,254 clean reads were obtained from Illumina high-throughput sequencing of small RNA libraries at 24 °C and 37 °C, respectively. We identified 149 conserved and 141 novel miRNAs. Seven conserved miRNAs and 77 novel miRNAs were differentially expressed at different temperatures. Target genes for differentially expressed known and novel miRNAs were predicted and functionally annotated. Gene Ontology (GO) analysis showed that high-ranking miRNA target genes were involved in metabolic processes, responses to stress, and signaling, indicating that these high temperature-responsive miRNAs have functions in diverse gene regulatory networks. Spatial expression patterns of the miRNAs and their target genes were found to be expressed in shoot tip and base tissues by qRT-PCR. In addition, high temperature reduced viral titers in the shoot meristem tip, while negatively regulated miRNA-mediated target genes related to resistance disease defense and hormone signal transduction pathway were up-regulated in the *P. pyrifolia* shoot tip in response to high temperature. These results suggested that miRNAs may have important functions in the high temperature-dependent decrease of ASGV titer in in vitro-grown pear shoots.

**Conclusions:**

This is the first report of miRNAs differentially expressed at 24 °C and 37 °C in the meristem tip of pear shoots infected with ASGV. The results of this study provide valuable information for further exploration of the function of high temperature-altered miRNAs in suppressing viral infections in pear and other fruit trees.

**Electronic supplementary material:**

The online version of this article (doi:10.1186/s12864-015-2126-8) contains supplementary material, which is available to authorized users.

## Background

Plant microRNAs (miRNAs) and small interfering RNAs (siRNAs) are two major classes of non-coding small RNAs with 21–24 nucleotides in length that mediate RNA silencing pathways to regulate gene expression and innate immune response [1–4]. However, miRNA and siRNA are distinct from modes of biogenesis and defense. Specifically, miRNA is primarily generated from a single-stranded precursor that forms a self-complementary stem and loop structure, whereas siRNA is derived from double-stranded RNAs or exogenous viruses. In plants, most miRNAs are processed specifically by the Dicer-Like 1 (DCL1) protein, and in contarst, siRNAs are cleaved by DCL2, DCL3 and DCL4. After excision, mature miRNAs or siRNAs are loaded onto the Argonaute (AGO) proteins, with other factors to assemble the RNA-induced silencing complex (RISC), guiding miRNA/siRNA to pair with specific RNA targets to implement the translational repression or silencing [5–11]. siRNA function is primarily to detect and eliminate invading viruses by targeting the virus-derived single strand RNA (ssRNA). However, miRNAs species usually negatively regulate their target genes via translational inhibition or cleavage of target mRNAs and thus play a pivotal role in a wide variety of biological processes such as plant development, signal transduction, resistance pathways, as well as abiotic and biotic stresses [12–16]. Upon viral infection, miRNAs often exhibit differential expression profiles [17–19]. For instance, miRNAs in cotton plants are differentially regulated by infection with *Cotton leafroll dwarf virus* (CLRDV) and some CLRDV-induced symptoms may be correlated with the deregulation of miRNA and/or epigenetic networks [20]. In *N. benthamiana* and *Arabidopsis*, the expression of miR168 and AGO1 mRNA is up-regulated in response to infection by several plant viruses [21–24]. The same also holds true for *soybean mosaic virus* (strain G7)-infected soybean plants carrying the resistance gene *Rsv1* [19]. As AGO1 protein is a central component of the RISC in the miRNAs/siRNAs-mediated post-transcriptional gene silencing (PTGS) pathways [25], these findings suggest the possible role of miRNA in regulating the innate antiviral silencing pathways in plants.

Pear is an important fruit tree crop cultivated worldwide. China, the world’s major producer of pears, has distinctive local pear varieties; however, many pear cultivars are commonly infected with *Apple stem grooving virus* (ASGV) and *Apple chlorotic leaf spot virus* (ACLSV), and viral infection dramatically reduces fruit quality [26–29]. Obtaining virus-free seedlings by heat treatment combined with shoot meristem tip culture is an effective way to control virus diseases in fruit trees [30]. In a previous study, we found that viruses were distributed unevenly in in vitro*-*grown pear shoots, and that the viral titer apparently decreased in the pear shoot apical meristem in response to high temperature treatment [31]. However, the molecular mechanism underlying the thermotherapy elimination of viruses is not known. To understand the relationship between the high temperature-dependent decrease in viral titer and the miRNA pathways in pear shoots, we used in vitro-grown pear shoots infected with ASGV, a member of the genus *Capillovirus* [32], as a research material. We sequenced and compared small RNAs prepared from *P. pyrifolia* shoot meristem tip tissue cultured in vitro at 24 °C and at 37 °C, a high temperature treatment. The expression levels of viral genomic RNA, miRNAs and mRNAs of their predicted target genes in the shoot meristem tip and base tissues were analyzed to explore the possible roles of miRNA regulation in the high temperature-dependent decrease in virus titer.

## Results

### Analysis of small RNAs from in vitro-cultured pear shoots infected with ASGV in response to high temperature

To identity miRNAs associated with high temperature treatment, small RNA differential expression libraries were constructed from 24 °C- and 37 °C-treated in vitro-grown pear shoots infected with ASGV and sequenced using high-throughput Solexa sequencing. After removing the low quality reads, 5′ primer contaminants, reads without the 3′ primer, reads with no insert tags, reads containing poly A tags, and reads shorter than 18 nt and longer than 30 nt reads, a total of 22,592,997 and 20,411,254 clean reads were obtained from the meristem tips of in vitro pear shoots cultured at 24 °C and 37 °C, respectively (Additional file 1). The sequenced clean small RNAs included different categories of exon antisense and sense, intron antisense and sense, rRNA, repeats, tRNA, snRNA, snoRNA, miRNA and other unannotated reads, of which miRNA tags accounted for 9,547,708 (42.26 %) and 11,115,138 (54.46 %) for the 24 °C and 37 °C libraries, respectively (Table 1), indicating that the proportion of miRNAs in the 37 °C library was higher than in the 24 °C library.

**Table 1:** Distribution of small RNA sequences among the different categories in the 24 °C and 37 °C treatment libraries constructed from in vitro-grown pear shoots

	T24		T37	
	Unique sRNA reads (percent %)	Total sRNA reads (percent %)	Unique sRNA reads (percent %)	Total sRNA reads (percent %)
Total	5529063 (100 %)	22592997 (100 %)	3974771 (100 %)	20411254 (100 %)
Exon antisense	30748 (0.56 %)	113906 (0.5 %)	28998 (0.73 %)	96547 (0.47 %)
Exon sense	50164 (0.91 %)	145765 (0.65 %)	46755 (1.18 %)	113202 (0.55 %)
Intron antisense	154430 (2.79 %)	498616 (2.21 %)	116982 (2.94 %)	3441841 (1.69 %)
Intron sense	264322 (4.78 %)	1148358 (5.08 %)	215436 (5.42 %)	856349 (4.2 %)
miRNA	20376 (0.37 %)	9547708 (42.26 %)	18595 (0.47 %)	11115138 (54.46 %)
rRNA	91764 (1.66 %)	1174482 (5.2 %)	92261 (2.32 %)	1337417 (6.55 %)
Repeat	2480 (0.04 %)	8235 (0.04 %)	1913 (0.05 %)	5485 (0.03 %)
snRNA	4025 (0.07 %)	10705 (0.05 %)	4470 (0.11 %)	13331 (0.07 %)
snoRNA	1365 (0.02 %)	2996 (0.01 %)	1591 (0.04 %)	3814 (0.02 %)
tRNA	7596 (0.14 %)	125805 (0.56 %)	9030 (0.23 %)	172127 (0.84 %)
Unannotated	4901793 (88.66 %)	9816421 (43.45 %)	3438740 (86.51 %)	6353660 (31.13 %)

**Fig. 1:**
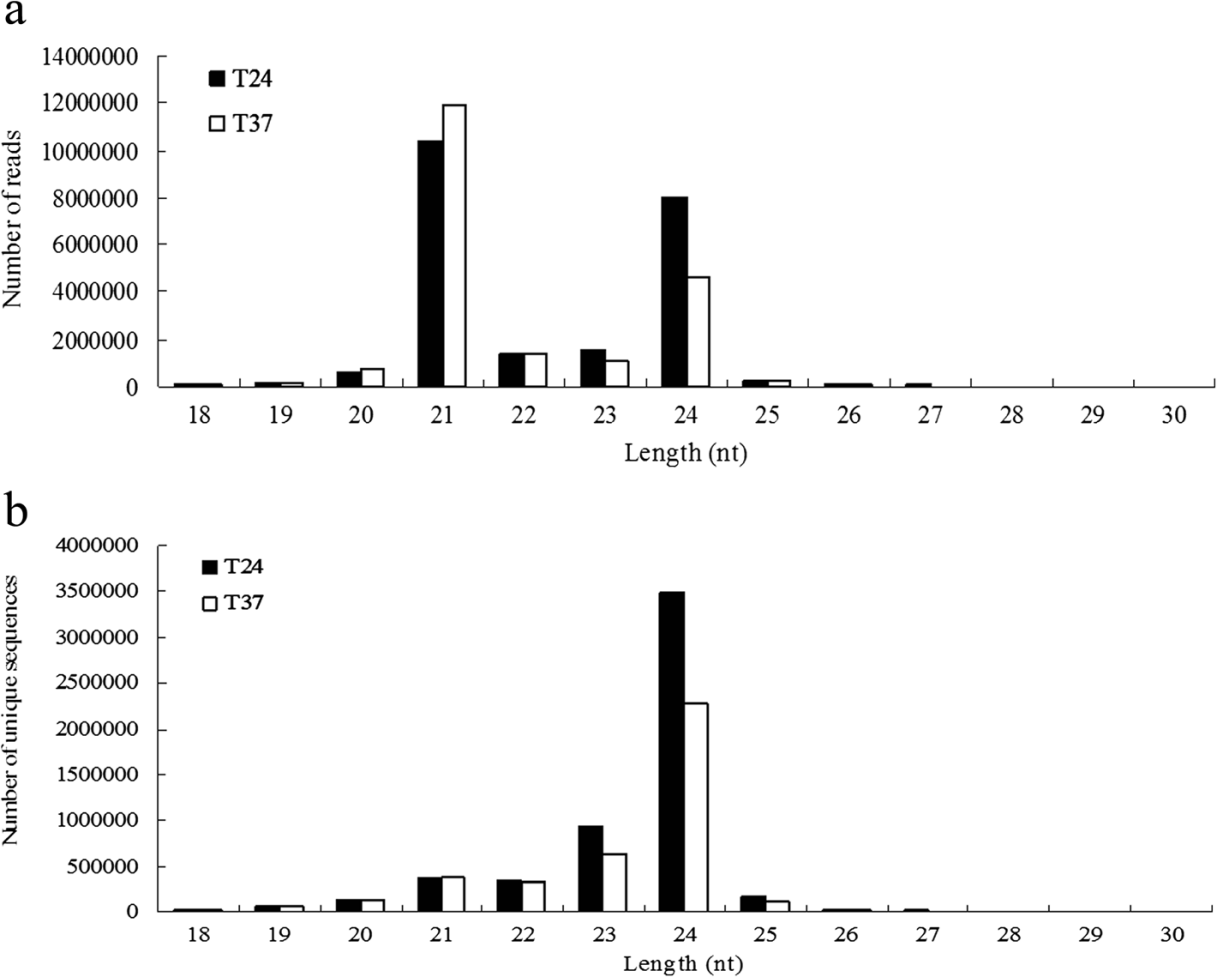
Length distribution of small RNA reads in the 24 °C and 37 °C libraries constructed from in vitro-grown shoots of *P. pyrifolia*. Length distribution of sRNA reads (**a**) and unique sequences (**b**) in the two libraries

**Fig. 2:**
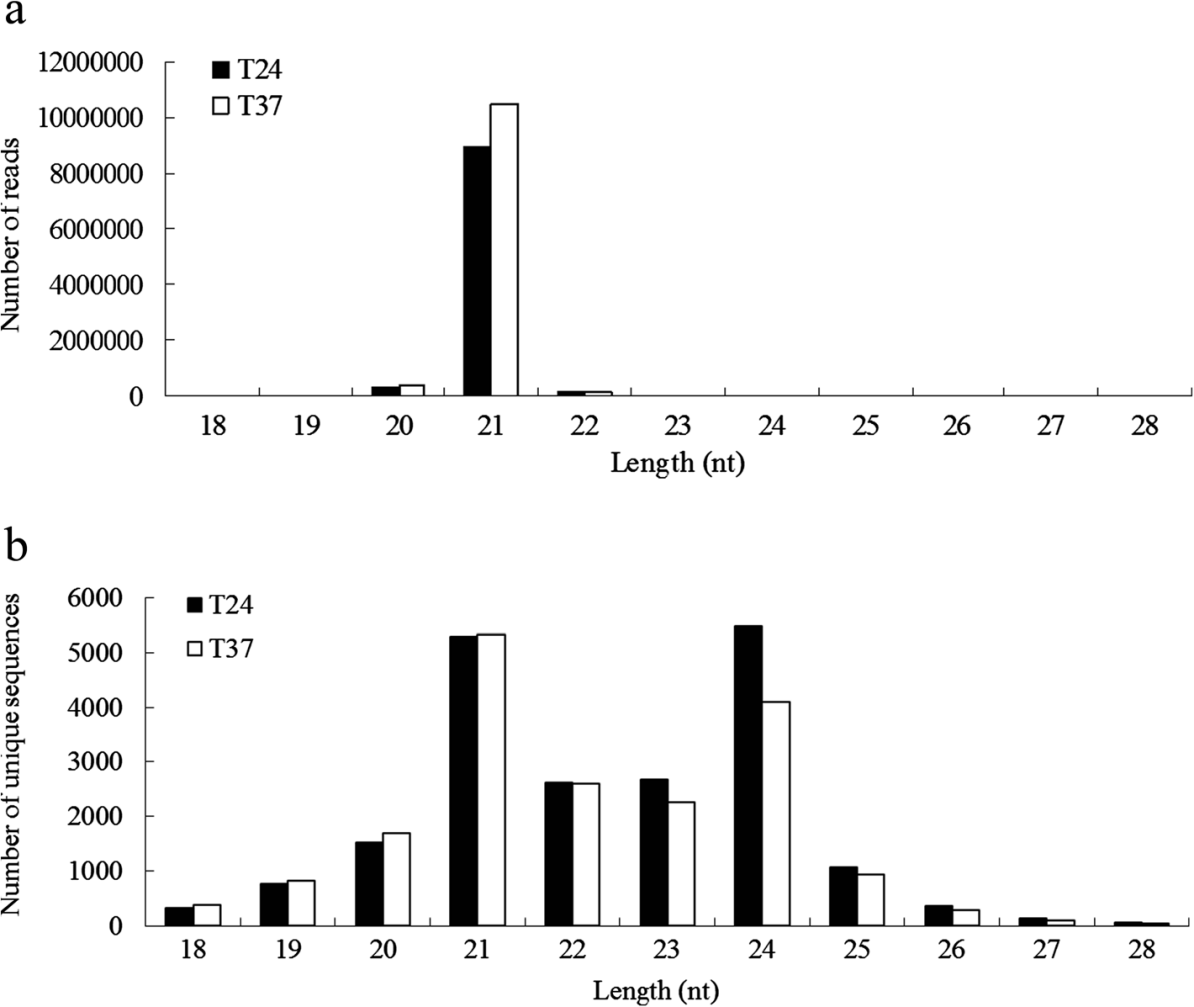
Characterization of miRNAs in the 24 °C and 37 °C in vitro pear shoot libraries. Length distribution of miRNA reads (**a**) and unique sequences (**b**) in the two libraries

Analysis of the length distribution of the small RNAs showed that the 21 nt molecules were the most abundant, with 10,360,625 (45.83 %) and 11,933,515 reads (58.4 %) in the 24 °C and 37 °C libraries, respectively. The next most abundant class was the 24 nt molecules, with 8,025,208 (35.5 %) and 4,646,604 reads (22.74 %) from the the 24 °C and 37 °C libraries (Fig. 1a). Interestingly, among the unique sequences, the 24 nt sRNA sequences were most abundant, followed by the 23 nt sRNA sequences, while the 21 nt and 22 nt sequences were present in similar amounts (Fig. 1b). Based on the sizes of miRNAs, the most abundant were 21 nt in length, with a total of 8,963,557 and 10,485,304 reads in the 24 °C and 37 °C libraries, respectively (Fig. 2a); this relative abundance of 21-nt miRNAs is similar to results reported previously in *P. mume* [33]. The majority of unique miRNA sequences fell in the range of 21–24 nt in length in both libraries (Fig. 2b), and among them, the 21-nt unique miRNAs were most abundant with 5285 and 5329 reads, followed by the 24-nt miRNAs with 5481 and 4104 reads, while 22-nt and 23-nt sequences were present in similar amounts in the 24 °C and 37 °C libraries, respectively .

Of the clean reads, the common and specific reads in the 24 °C and 37 °C libraries from the in vitro-grown pear plants, including the unique reads and total reads, were calculated (Addiitonal file 2). The results showed that 11.65 % total sRNAs (51.78 % unique sRNAs) with an average sequence mean frequency of 1.17 were specifically present in the 24 °C library, 6.9 % (32.92 % unique) with an average frequency of 1.09 were found only in the 37 °C library, and 81.45 % (15.3 % unique) with an average common sequence frequency of 27.78 were co-present in both libraries (Additional file 2). Taken together the above results suggested that there is a huge difference in the number of reads between the 24 °C and 37 °C samples.

### Characterization of known miRNAs in *P. pyrifolia*

**Fig. 3:**
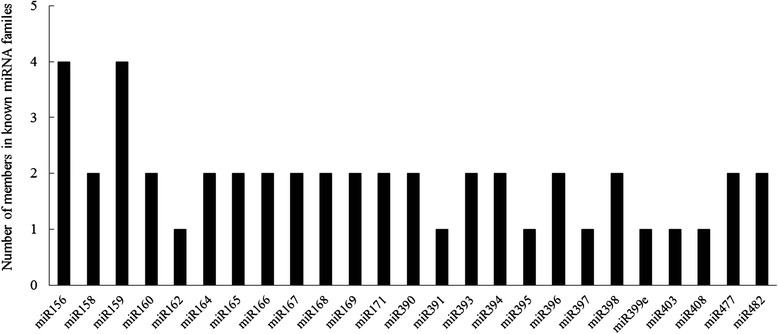
Number of miRNA members identified in each conserved miRNA family in the small RNA libraries

**Fig. 4:**
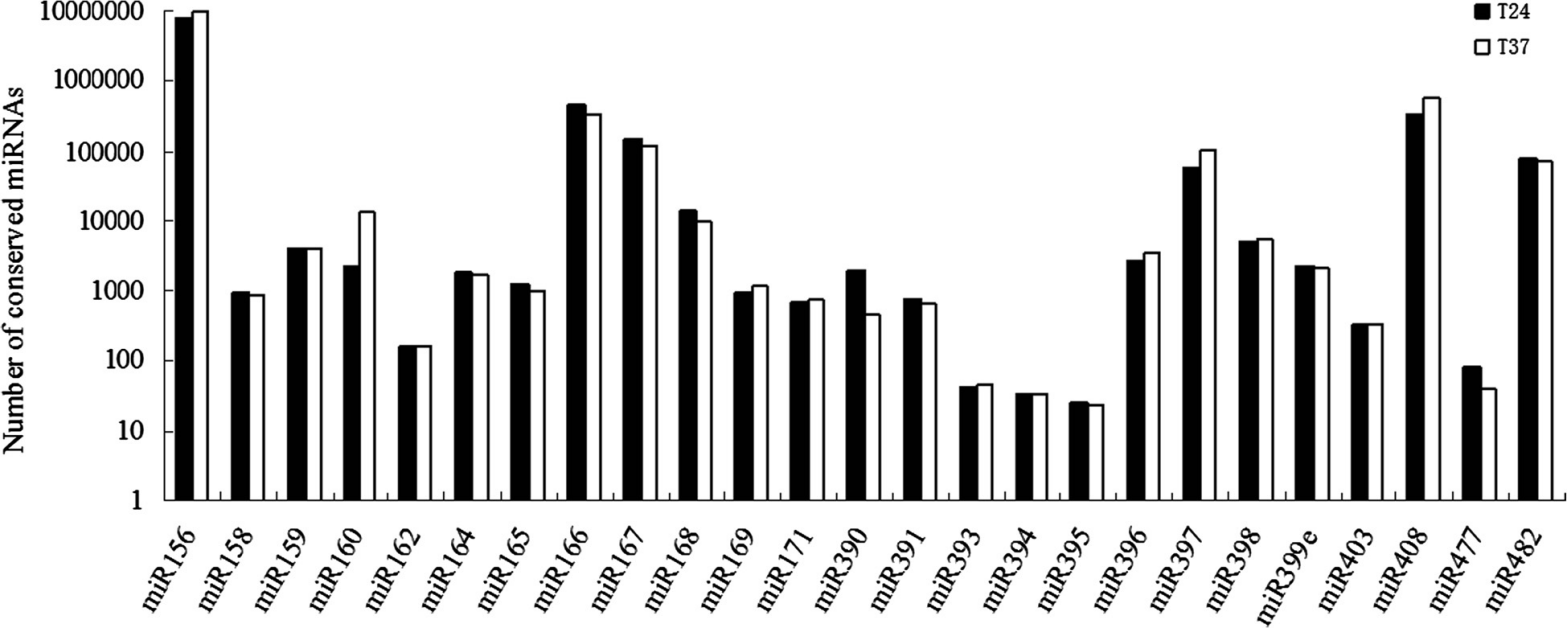
Count numbers of identified miRNAs in each conserved miRNA family in the 24 °C and 37 °C in vitro pear shoot libraries

Known miRNAs in *P. pyrifolia* were identified by alignment to a designated part of miRBase17.0. One hundred forty-nine known miRNAs in the two *P. pyrifolia* libraries were obtained and identified after removing miRNAs in which the expression levels were too low to be analyzed for differential expression (Additional file 3). Forty-seven members, belonging to 25 conserved miRNA families, were found in the two *P. pyrifolia* libraries. The numbers and the read counts for the conserved miRNA families were analyzed (Figs. 3 and 4). A majority of the 25 miRNA families contained 2 members, families miR156 and miR159 each contained 4 members, and the remaining ones including miR162, miR391, miR395, miR397, miR399e, miR403, and miR408 had only one member each. The most abundant miRNA was miR156 with 8,192,170 and 9,689,927 reads in the 24 °C and 37 °C libraries from *P. pyrifolia*, respectively, while miR156, miR157, miR397, and miR408 were moderately abundant. These results showed that the miRNA members exhibited significant differential expression levels, in agreenment with findings from other fruit species such as apple and grape [34, 35]. In addition to the conserved miRNAs, 102 non-conserved miRNAs were identified, of which miR535 was the most abundant with 438,839 and 413,421 reads in the 24 °C and 37 °C libraries, respectively (Additional file 3).

### Identification of novel candidate miRNAs in *P. pyrifolia*

The characteristic hairpin structure of miRNA precursors or detection of the corresponding miRNAs can be used to identify novel miRNAs. Beijing Genomics Institute (BGI) company (Shenzhen city, China) has developed a prediction software, Mireap to predict novel miRNAs by exploring the secondary structure using the mfold web server, the Dicer cleavage sites, and the minimum free energy of the annotated small RNA tags which could be mapped to the pear genome sequence (http://peargenome.njau.edu.cn) [36–40]. A total of 630 new miRNAs were identified, of which 491 and 530 miRNA candidates were from the 24 °C and 37 °C libraries, respectively. After removing those miRNAs with expression levels that were too low to be analyzed for differential expression, 141 novel miRNAs were identified, of which 77 were differentially expressed; novel262 had the highest abundance with 833 transcripts per million (TPM), followed by novel345 with 43 TPM in the 24 °C library. In the 37 °C library, novel19 had the highest abundance at 30 TPM, followed by novel337 and novel540 with 18 and 10 TPM, respectively (Table 2). The results from this analysis revealed that most of the novel miRNAs were present in relatively low abundance, as indicated by their frequencies, in comparison with the conserved miRNAs. In addition, among the 141 novel miRNAs, 32 had complementary miRNA*s, with precursor lengths ranging from 83 to 349 nt and predicted minimal folding energy (MFE) ranging from −28.6 to −161.4 kal/mol (Additional file 4, Additional file 5, and Additional file 6). This is in agreement with published criteria for novel miRNA [36–38], and indicates that these candidate miRNAs are most likely to be new miRNA family members in *P. pyrifolia*. Twenty out of 32 new miRNA/new family members were 21 nt in length, while four, seven, and one miRNA had lengths of 23, 22, and 20 nt, respectively (Additional file 4). Also, 109 miRNAs without miRNA*s detected were identified as candidate miRNAs (Additional file 7); these loci, pre-miRNA sequences and structures, and reads from deep sequencing were also shown in Additional file 6 and Additional file 7. The base bias in the first position among the predicted novel miRNA candidates showed that the majority of these novel miRNA candidates started with a 5′ uridine (U) as shown in Additional file 8. In addition, novel miRNA candidate nucleotide bias at each position were also analyzed (Additional file 9).

**Table 2:** miRNAs expressed differentially between the 24 °C and 37 °C treatments from in vitro-grown shoots of *P. pyrifolia*

miRNA name	T24		T37		Fold-change log_2_(T37/T24)	P-value	Mode	Sig-lable
	Counts	Normalized	Counts	Normalized				
miR1043-3p	43	1.9	16	0.78	−1.28	0.001	Down	**
miR3627-5p	368	16.29	1827	89.51	2.46	2.29e-265	Up	**
miR397a	56569	2503.83	104360	5112.87	1.03	0	Up	**
miR477b	38	1.68	13	0.64	−1.4	0.001	Down	**
miR4993	1677	74.23	749	36.7	−1.02	5.55e-62	Down	**
miR5519	18	0.8	34	1.67	1.06	0.01	Up	**
miR5801	90	3.98	40	1.96	−1.02	0.0001	Down	**
novel124	45	1.99	18	0.88	−1.18	0.003	Down	**
novel125	30	1.33	0	0.01	−7.05	4.32e-09	Down	**
novel147	28	1.24	12	0.59	−1.07	0.02	Down	*
novel160	28	1.24	58	2.84	1.20	0.0002	Up	**
novel166	39	1.73	0	0.01	−7.43	1.32e-11	Down	**
novel168	85	3.76	0	0.01	−8.56	1.82e-24	Down	**
novel177	118	5.22	0	0.01	−9.02	1.09e-33	Down	**
novel188	42	1.86	0	0.01	−7.54	1.91e-12	Down	**
novel19	214	9.5	611	29.93	1.66	2e-54	Up	**
novel190	45	1.99	0	0.01	−7.64	2.77e-13	Down	**
novel197	27	1.2	10	0.49	−1.29	0.01	Down	*
novel202	31	1.37	0	0.01	−7.1	2.27e-09	Down	**
novel203	139	6.15	0	0.01	−9.26	1.46e-39	Down	**
novel228	11	0.49	25	1.22	1.33	0.008	Up	**
novel233	12	0.53	22	1.08	1.02	0.04	Up	*
novel241	145	6.42	0	0.01	−9.33	3.08e-41	Down	**
novel246	31	1.37	5	0.25	−2.49	2.84e-05	Down	**
novel247	29	1.28	0	0.01	−7	8.22e-09	Down	**
novel250	11	0.49	23	1.13	1.21	0.02	Up	*
novel26	24	1.06	0	0.01	−6.73	2.05e-07	Down	**
novel262	18817	832.87	0	0.01	−16.35	0	Down	**
novel266	327	14.47	0	0.01	−10.5	4.1e-92	Down	**
novel27	37	1.64	0	0.01	−7.36	4.77e-11	Down	**
novel281	25	1.11	11	0.54	−1.04	0.04	Down	*
novel290	57	2.52	0	0.01	−7.98	1.22e-16	Down	**
novel306	18	0.8	42	2.06	1.37	0.00046	Up	**
novel308	29	1.28	0	0.01	−7	8.22e-09	Down	**
novel312	26	1.15	0	0.01	−6.85	5.67e-08	Down	**
novel337	131	5.8	364	17.83	1.62	4.89e-32	Up	**
novel338	16	0.71	29	1.42	1.004	0.02	Up	*
novel345	961	42.53	0	0.01	−12.05	2.43e-269	Down	**
novel348	35	1.55	0	0.01	−7.28	1.73e-10	Down	**
novel363	7	0.31	24	1.18	1.92	0.00078	Up	**
novel379	0	0.01	34	1.67	7.38	9.41e-12	Up	**
novel385	0	0.01	48	2.35	7.88	2.77e-16	Up	**
novel393	0	0.01	38	1.86	7.54	4.78e-13	Up	**
novel395	0	0.01	48	2.35	7.88	2.77e-16	Up	**
novel412	0	0.01	84	4.12	8.68	6.19e-28	Up	**
novel413	0	0.01	39	1.91	7.58	2.27e-13	Up	**
novel421	0	0.01	21	1.03	6.69	1.52e-07	Up	**
novel423	0	0.01	44	2.16	7.75	5.46e-15	Up	**
novel429	0	0.01	23	1.13	6.82	3.42e-08	Up	**
novel438	0	0.01	116	5.68	9.15	2.72e-38	Up	**
novel439	0	0.01	22	1.08	6.75	7.2e-08	Up	**
novel447	0	0.01	42	2.06	7.68	2.42e-14	Up	**
novel457	0	0.01	33	1.62	7.34	1.98e-11	Up	**
novel476	0	0.01	94	4.61	8.85	3.59e-31	Up	**
novel482	0	0.01	165	8.08	9.66	3.77e-54	Up	**
novel497	0	0.01	22	1.08	6.75	7.2e-08	Up	**
novel498	0	0.01	41	2.01	7.65	5.11e-14	Up	**
novel504	0	0.01	49	2.4	7.9	1.32e-16	Up	**
novel509	0	0.01	67	3.28	8.36	1.97e-22	Up	**
novel516	0	0.01	30	1.47	7.2	1.85e-10	Up	**
novel52	35	1.55	15	0.73	−1.08	0.01	Down	**
novel526	0	0.01	142	6.96	9.44	1.05e-46	Up	**
novel531	0	0.01	40	1.96	7.61	1.08e-13	Up	**
novel533	0	0.01	26	1.27	6.99	3.65e-09	Up	**
novel540	0	0.01	216	10.58	10.05	1.18e-70	Up	**
novel543	0	0.01	24	1.18	6.88	1.62e-08	Up	**
novel558	0	0.01	22	1.08	6.75	7.2e-08	Up	**
novel560	0	0.01	22	1.08	6.75	7.2e-08	Up	**
novel564	0	0.01	24	1.18	6.88	1.62e-08	Up	**
novel566	0	0.01	30	1.47	7.2	1.85e-10	Up	**
novel573	0	0.01	25	1.22	6.94	7.7e-09	Up	**
novel579	0	0.01	41	2.01	7.65	5.11e-14	Up	**
novel585	0	0.01	32	1.57	7.29	4.18e-11	Up	**
novel586	0	0.01	24	1.18	6.88	1.62e-08	Up	**
novel598	0	0.01	37	1.81	7.5	1.01e-12	Up	**
novel6	224	9.91	82	4.02	−1.3	1.5e-13	Down	**
novel601	0	0.01	26	1.27	6.99	3.65e-09	Up	**
novel603	0	0.01	66	3.23	8.34	4.14e-22	Up	**
novel615	0	0.01	118	5.78	9.18	6.13e-39	Up	**
novel69	842	37.27	372	18.23	−1.03	9.68e-33	Down	**
novel8	31	1.37	58	2.84	1.05	0.00082	Up	**
novel83	24	1.06	0	0.01	−6.73	2.05e-07	Down	**
novel84	27	1.2	0	0.01	−6.9	2.98e-08	Down	**
novel99	23	1.02	0	0.01	−6.67	3.91e-07	Down	**

* represnt Fold-change(log T37/T24) >1.0 or Fold-change(log T37/T24) <-1.0, and 0.01<=P -values <0.05; ** represnt Fold-change(log T37/T24) >1.0 or Fold-change(log T37/T24) <-1.0, and P -values <0.01

### Changes in differential abundance levels of miRNAs in response to high temperature in *P. pyrifolia*

To identify high temperature-responsive miRNAs, the normalized expression of miRNAs in the two libraries constructed from the 24 °C and 37 °C treatments was compared. The miRNAs that showed changes in expression levels >1.0-fold with *p-values* less than 0.05 in response to high temperature treatment in *P. pyrifolia* infected with ASGV were presented in Table 2. The results showed that seven known miRNA candidates were differentially expressed, while 77 potentially new miRNA candidates were differentially expressed between the two libraries. Three known miRNAs and 47 novel miRNAs were up-regulated, while four known miRNAs and 30 novel miRNAs were down-regulated in response to 37 °C treatment. Among the 77 differentially expressed potentially novel miRNAs/new members, 37 novel miRNA were expressed specifically in response to 37 °C, while 22 novel miRNAs were specifically expressed in the 24 °C library (Table 2).

**Fig. 5:**
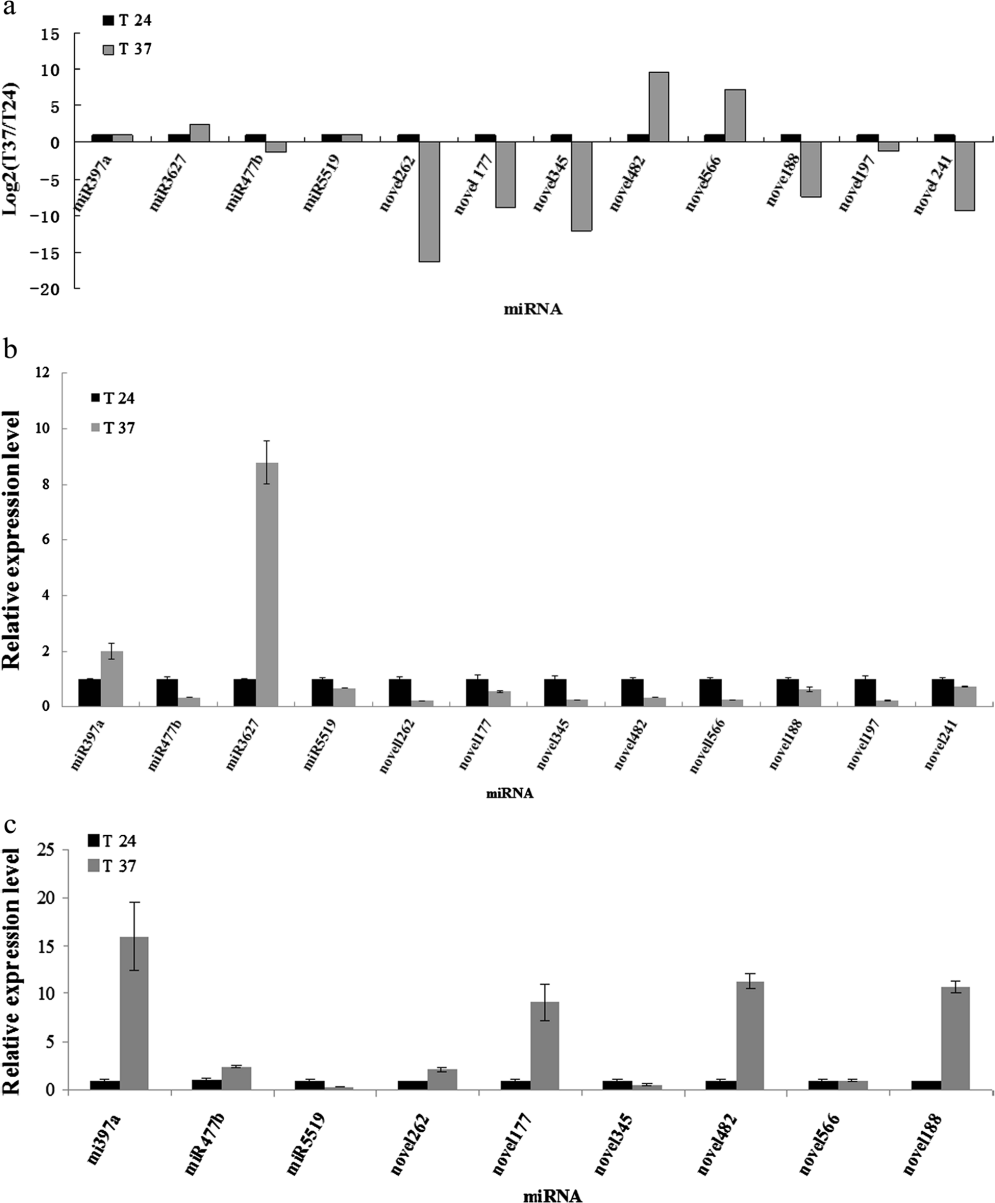
Relative miRNA expression profiles determined in in vitro pear shoot tips in the 24 °C and 37 °C libraries by deep sequencing (**a**) and quantitative real-time PCR (**b**), and in base tissue by qRT-PCR (**c**)

To validate the existence of the predicted differential expression of miRNAs in the pear shoot meristem tip tissue, four known and eight novel miRNAs that were differentially expressed in the two libraries were analyzed by poly (A) real-time quantitative PCR. The PCR primers were listed in Additional file 10. Gene expression levels were presented as fold-changes in the 37 °C treatment shoots relative to expression in the 24 °C shoots. The results showed that the expression patterns of four known miRNAs and eight novel miRNAs determined by qRT-PCR were similar to those from the deep sequencing data except for miRNA5519, novel482, and novel566 (Fig. 5a, b). Results from deep sequencing and real-time PCR demonstrated that 37 °C high temperature-responsive miR397a and miR3627-5p were up-regulated in the shoot meristem tip tissue, while miR477b, novel262, novel177, novel345, novel188, novel197, and novel241 were down-regulated in response to 37 °C treatment (Fig. 5a, b).

To investigate the effect of high temperature on viral titer with respect to miRNA pathways in different tissues, the expression levels of miR397a, miR477b, miR3627-5p, miR5519, novel262, novel177, novel345, novel482, novel566, novel188, novel197, and novel241 were also quantified in the shoot basal tissues treated at 24 °C and 37 °C using qRT-PCR. The results showed that the 37 °C treatment induced 2-16 fold increases in miR397a, miR477b, novel262, novel177, novel482, and novel188 expression levels in shoot basal tissue. Treatment at 37 °C also resulted in a 0.32-fold decrease in miR5519 and a 0.5-fold decrease in novel345 expression levels, with no apparent fold-change in novel566 (Fig. 5c). The relative expression levels of four miRNAs (miR477b, novel262, novel177, and novel188) changed significantly in both tissues but in opposite directions. Two miRNAs (miR397a and novel345) changed significantly in the base as compared to the tip (Table 3). Thus, our results show that miRNAs in pear shoots cultured in vitro have different expression profiles in the shoot meristem tip as compared to base tissues, indicating that miRNAs differentially regulated at high temperature exhibit tissue-specific expression patterns.

**Table 3:** Expression patterns of differentially expressed miRNAs and targeted mRNA genes analyzed in meristem tip and base tissue of in vitro-grown pear shoots by qRT-PCR and deep sequencing

ID miRNA	Log_2_(T37/T24)	q RT-PCR		ID target gene	q RT-PCR		Functional annotation
	Tip	Tip	Base		Tip	Base	
miR397a	Up (1.03)	Up (2.0)	Up (15.9)	Pbr0359622	Down (0.15)	Up (1.22)	Laccase-4-like
miR477b	Down (−1.4)	Down (0.3)	Up (2.39)	Pbr027204	Up (4.43)	-	DELLA protein RGL1-like
miR3627-5p	Up (2.46)	Up (8.8)	-	--	--	--	--
miR5519	Up (1.06)	Down (0.67)	Down (0.32)	Pbr002489	Up (1.32)	-	Proteasome subunit alpha type-4
novel262	Down (−16.35)	Down (0.22)	Up (2.1)	Pbr030437	Up (1.23)	Up (1.47)	ABC transporter A family member
novel177	Down (−9.02)	Down (0.55)	Up (9.1)	Pbr019211	Up (3.66)	Up (2.64)	NAC domain-containing protein 72-like
novel345	Down (−12.05)	Down (0.25)	Down (0.5)	Pbr042600	Up (1.8)	Up (1.04)	2-oxoglutarate dehydrogenase, mitochondrial-like
novel482	Up (9.66)	Down (0.33)	Up (11.3)	Pbr025376	Up (1.42)	Down (0.37)	disease resistance protein RGA2-like
novel566	Up (7.2)	Down (0.46)	Down (0.99)	Pbr023226	Down (0.59)	Up (1.32)	E3 ubiquitin-protein ligase UPL1-like
novel188	Down (−7.54)	Down (0.63)	Up (10.7)	Pbr017710	Up (2.4)	Up (2.6)	Squamosa promoter-binding-like Protein13A
novel197	Down (−1.21)	Down (0.22)	-	Pbr039336	-	-	probable methyltransferase PMT24
novel241	Down (−9.33)	Down (0.74)	-	Pbr004679	-	-	probable mannitol dehydrogenase

“-” indicate no detected data by real-time PCR; “--” indicates that miR3627-5p had no predicted target gene

### Prediction of potential targets of differentially expressed miRNAs in *P. pyrifolia*

**Fig. 6:**
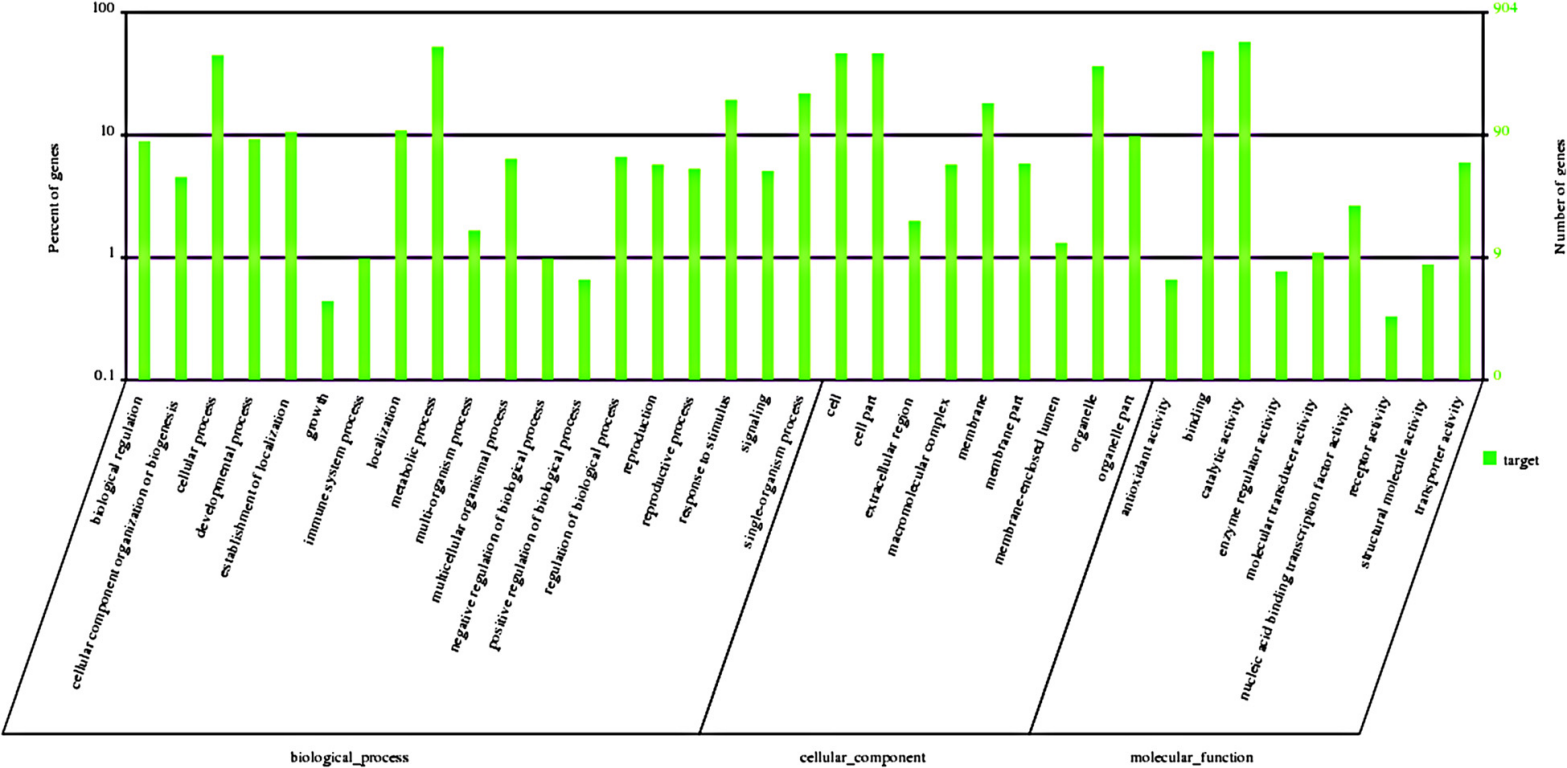
Gene ontology (GO) analysis of the predicted targets for the differentially expressed miRNAs

Hundreds of putative target genes of differentially expressed miRNAs that responded to high temperature treatment in pear were predicted by bioinformatics analysis. Our results showed that *P. pyrifolia* miRNA targets encoded not only indispensible transcription factors, but also non-transcription factor proteins involved in diverse physiological processes. To investigate the regulatory function of miRNAs in *P. pyrifolia* infected with ASGV, hundreds of potential target genes for four conserved and 64 novel differentially-expressed miRNAs were predicted by Gene Ontology (GO) analysis. There are three structured ontologies in GO as followings: biological processes, cellular component, and molecular function (Fig. 6). In biological processes, the genes were classified into 19 categories. The top five over-represented GO terms were metabolic process, cellular process, single-organism process, response to stimulus, and signaling. Nine cellular components were identified, with the top four being cell, cell part, organelle, and membrane. Nine molecular functions were identified, with the most frequent being catalytic activity, binding, transporter activity, and nucleic acid binding transcription factor activity. These results indicated that the miRNAs have broad functions in gene regulatory networks. In addition, many miRNAs may regulate target genes that are involved in disease resistance and defense. For example, the expression of the putative disease resistance protein targeted by novel166, was up-regulated in response to 37 °C treatment by RNA-seq analysis (unpublished). High temperature treatment (37 °C) specifically induced expression of novel476 and novel482 miRNAs that target a disease resistance RPP13-like protein and disease resistance protein RGA2-like, respectively. This indicates that the predicted miRNA target genes have important functions in the response to high temperature; however the mechanism behind the miRNA-mediated high temperature reduction in viral titer is presently unknown.

### The effect of high temperature on ASGV in the shoot meristem tip tissue of in vitro-grown *P. pyrifolia*

**Fig. 7:**
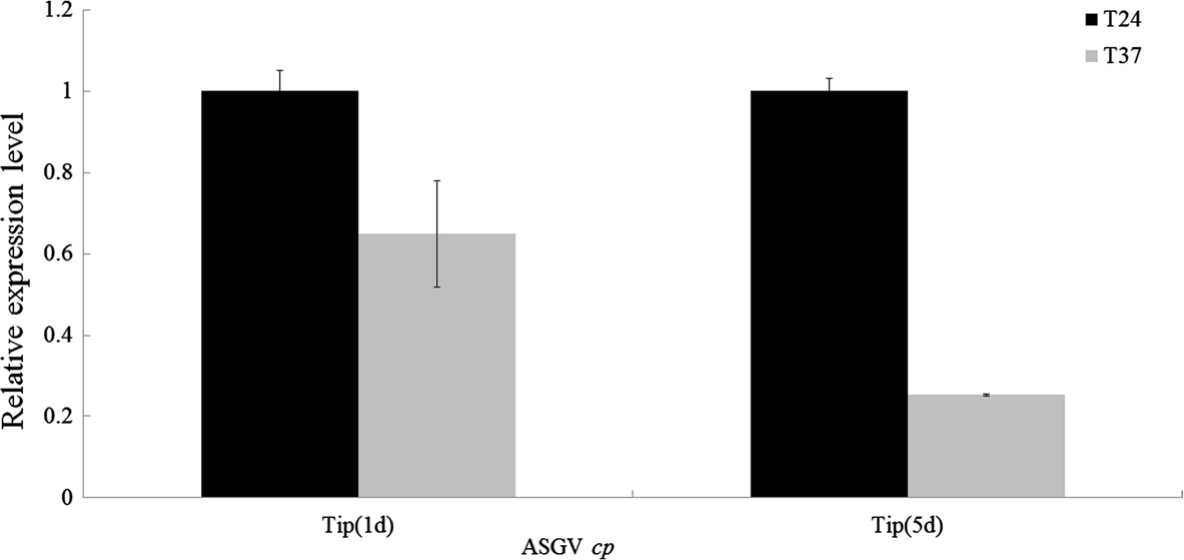
ASGV virus titer in tip meristem tissues of in vitro pear shoots determined by qRT-PCR analysis of the ASGV *cp* gene in the 24 °C and 37 °C small RNA libraries

To investigate the effects of high temperature on ASGV accumulaiton in in vitro-grown *P. pyrifolia* shoot meristem tip tissues, the ASGV genomic RNA in the samples treated at 24 °C (normal growth temperature) and 37 °C (high temperature; thermotherapy) was quantified by real-time PCR to detect the *cp* gene. It was found that in comparison with the control, ASGV was reduced to 0.6-fold and to 0.25-fold after 37 °C treatment for 1 and 5days, respectively (Fig. 7). Apparantly, the ASGV titer in the meristem tip tissue decreased along with increasing treatment times at high temperature.

### Spatial expression patterns of potential miRNA-mediated target genes in response to high temperature in *P. pyrifolia* shoots

**Fig. 8:**
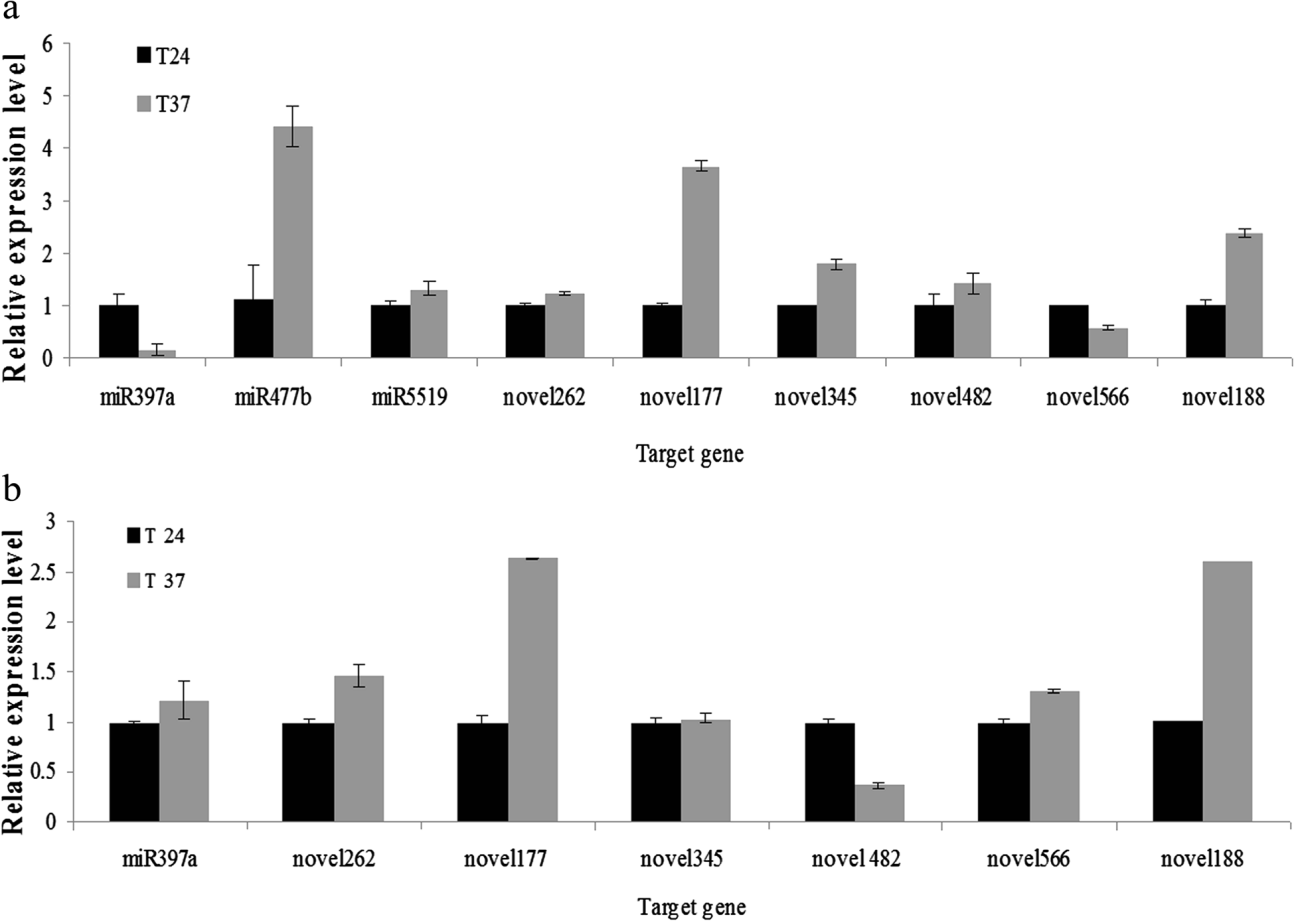
miRNA target gene expression profiles in the shoot tip (**a**) and base tissues (**b**) of in vitro-grown pear shoots determined by qRT-PCR

To examine if high temperature-responsive miRNAs can regulate the expression of their target genes, the expression levels of selected miRNAs and their target mRNAs were analyzed from tip and base tissues of pear shoots. The changes in spatial expression patterns of laccase-4-like, DELLA protein RGL1-like, proteasome subunit alpha type-4, an ABC transporter A family member, NAC domain-containing protein 72-like, 2-oxoglutarate dehydrogenate, mitochondrial-like, disease resistance protein RGA2-like, E3 ubiquitin-protein ligase UPL1-like, and squamosa promoter-binding-like Protein13A, as predicted targets of miR397a, miR477b, miR5519, novel262, novel177, novel345, novel482, novel566, and novel188, respectively, were quantified using qRT-PCR (Fig. 8). The correlations between the expression levels of these miRNAs and their target mRNAs in pear shoot tip and base tissues in response to high temperature were shown in Table 3.

In shoot apices, negative correlations were observed for miR397a, miR477b, miR5519, novel262, novel177, novel345, novel482, and novel188 and their corresponding putative target genes, except for novel566. Putative target gene DELLA protein RGL1-like (targeted by miR477b), proteasome subunit alpha type-4 (by miR5519), ABC transporter A family member (by novel262), NAC domain-containing protein 72-like (by novel177), 2-oxoglutarate dehydrogenate, mitochondrial-like (by novel345), disease resistance protein RGA2-like (by novel482), and squamosa promoter-binding-like Protein13A (by novel188) were all up-regulated with relative quantitive (RQ) values of 4.43,1.32, 1.23, 3.66, 1.8, 1.42, and 2.4 in the 37 °C treatment, respectively. We also analyzed expression patterns of several miRNA-mediated target genes in shoot base tissue. The results showed that the expression levels of target gene Laccase-4-like increased at 37 °C, with RQ 1.22. Expression of ABC transporter A family member and NAC domain-containing protein 72-like also increased at 37 °C, with RQ values of 1.47 and 2.64, respectively. mRNA levels of 2-oxoglutarate dehydrogenase and squamosa promoter-binding-like Protein13A at 37 °C also increased 1.04 and 2.6-fold, respectively. This shows that the changes in mRNA levels in stem base tissue and in shoot tip tissue are different. In addition, the other target genes showed the opposite expression patterns in base tissue compared with tip tissues (Fig. 8 and Table 3).

## Discussion

### High-throughput sequencing of *P. pyrifolia* small RNAs

In this study, millions of small RNA reads from libraries prepared from ASGV-infected *P. pyrifolia* shoots grown at 24 °C and 37 °C were obtained by high-throughput sequencing. The pear genome sequence, released in 2012, was used for annotating the specific miRNAs of *P. pyrifolia* [40]. The most abundant small RNA reads in the two libraries prepared from the *P. pyrifolia* samples were 21 nt in length, followed by 24 nt, while 24 nt reads were predominant in the unique small RNA reads (Figs. 1 and 2). This is different from reports in other plant species in which 24 nt sRNAs are more abundant, such as in the wheat response to powdery mildew infection and heat stress [41, 42]. The ratio of sRNA total reads/unique sequences in the 24-nt class was 2.3 and 2 in the 24 °C and 37 °C libraries, respectively, while the ratios in the 21-nt class were 29 and 32 in the 24 °C and 37 °C libraries, respectively. The length distribution patterns of the sRNA read may refelct the compositions of the small RNA sample. For example, miRNAs are normally 21 or 22 nt long, siRNAs are 24 nt, and piRNAs are between 28 and 30 nt in length. We observed that the 21-nt small RNAs increased and the 24-nt small RNAs decreased in abundance in response to 37 °C treatment, and the 21 nt miRNAs were more abundant in the 37 °C library than in the 24 °C library. These observations demonstrate that the expression of miRNAs and siRNAs is significantly altered by the high temperature treatment, and such dynamic small RNA profiles may have important functions in suppressing viral infection under high temperature treatment.

### Possible functional roles of miRNAs in suppressing viral infection in high temperature-treated *P. pyrifolia*

In the *P. pyrifolia* shoot meristem tip, seven known and 77 potentially novel miRNAs were shown to be differentially expressed in response to high temperature (37 °C) treatment and the predicted target genes of these differentially expressed miRNAs were also identified (Table 2 and Fig. 6). These datas provide valuable insight information about the complex miRNA-mediated regulatory networks in pear shoot meristem tips infected with ASGV in response to high temperature treatment. To the best of our knowledge, this is the first report of high temperature-altered miRNAs that are involved in viral infection.

miRNAs may play important roles in host resistance against abiotic stress and biotic pathogens. For example, down-regulation of miR398 in *A. thaliana* up-regulates its target gene *CSD* encoding Cu/Zn superoxide dismutase, a known enzyme that is related to defense response against *Pseudomonas syringae* [3, 16, 43, 44]. In addition, it has also been reported that infections by *Tobacco mosaic virus* and *Potato virus Y* lead to changes in miRNA expression levels that affect the ability of plants to resist disease. These previous studies have demonstrated that miRNAs can regulate key genes in disease resistance pathways to affect viral infections [13, 17, 18, 45, 46]. In this study, we identified a number of predicted miRNA-target genes from *P. pyrifolia* that are possibly involved in disease resistance and defense in the high temperature-induced reduction in ASGV titers (Fig.6 and Table 3). For instance, Laccase targeted by miR397a is involved in the formation of the plant cell wall, and plays an important role in the defense response [47]. DELLA protein RGL1-like, TIR-NBS-LRR, and RGA2-like predicted to be targeted by miRNA477b, novel421 and novel482, respectively are also known as disease resistance proteins. Interestingly, we found that the targets of novel177 severely down-regulated in high temperature include NAC transcription factor, heat shock factor protein HSF8-like, and E3 ubiquitin-protein ligase COP1-like, which suggests that novel177 is involved in a complicated miRNA regulatory network. The computational analysis combined with the qRT-PCR results showed that NAC is negatively regulated by novel177 (Figs. 5 and 8). Numerous members of the NAC gene family have been shown to respond to biotic and abiotic stresses which may activate the plant defense response [48–52]. In this study, we found that significantly repressed expression of novel177 significantly up-regulated NAC expression in the shoot meristem tip of *P. pyrifolia* with low ASGV titer at 37 °C as compared to 24 °C (Fig. 8 and Table 3). In *Arabidopsis,* NAC1, regulated by miRNA164, is crucial for the formation of boundaries between meristems and emerging organ primordia, and affects auxin metabolism in the auxin signaling pathway [53–55]. It is possible that up-regulated NAC accelerates the growth and developmental process of the shoot meristem tip tissue, and inhibits the moevment of ASGV into the newly divided tip cells in an as-yet unknown manner during high temperature, leading to the reduced viral titer in the shoot meristem tip [56]. Based on above disucssion, it is very likely that the mixed action of these miRNA-mediated target genes contributes to the thermo-elimination of ASGV in pear. Indeed, high temperature can activate many highly conserved biological and adaptive responses that are regulated by miRNAs, which exhibit protective functions for the induction of the disease resistance response in plants [41, 56–58]. Future work is aimed at elucidation of the mechanistic details of these miRNA-regulated defense genes in inibiting viral infection at high temperature.

## Conclusions

This is the first report of miRNAs differentially expressed at 24 °C and 37 °C in the meristem tip of in vitro-grown pear shoots infected with ASGV. Using Illumina high-throughput sequencing, the numbers and types of miRNAs were systematically identified from in vitro-grown *P. pyrifolia* shoot meristem tip tissue at 24 °C and 37 °C. This was done in combination with qRT-PCR to explore the interaction between miRNA-regulated pear genes and ASGV in response to high temperature treatment. A total of seven known and 77 novel miRNAs were found to be expressed differentially in response to high temperature. Putative target genes were predicted and then annotated by GO databases to explore predicted gene functions. Comparative analysis of expression levels in relation to virus titer, miRNA and miRNA-mediated target gene expression patterns were performed to reveal the role of miRNA regulation on the decrease in virus titer from the shoot meristem tip observed under conditions of high temperature. Our research provided valuable information for further exploration of the function of these miRNAs/mRNA in associaiton with the reduced viral titer in meristem tip of *P. pyrifolia* shoots inducible to high temperature treatment.

## Methods

### Plant materials

*P. p*yrifolia cv. ‘Jinshui no. 2’, a widely grown cultivar in central and southern China, was used in the experiments. The establishment of in vitro *P. p*yrifolia cv. ‘Jinshui no. 2’ shoots in culture was performed as reported [59]. The presence of ASGV in each of the different explants was determined by reverse transcrption (RT)-PCR. In vitro shoots about 1 cm in length were transferred to freshly prepared MS medium and incubated in a thermotherapy chamber at 37 °C with a day/night regime of 16 h light at an intensity of 1500 lux and 8 h dark. Non-treated shoots infected with ASGV were kept at 24 °C as controls. Six shoots of 5 mm in length were collected from meristem tip and treated at 37 °C for 1 and 5 days, with three replications per treatment. Untreated meristem tip shoots (5 mm) maintained at 24 °C for 1 and 5 days were used as controls. All samples were frozen in liquid nitrogen immediately and stored at −80 °C prior to extraction of total RNA.

### Small RNA library construction and Illumina sequencing

To construct the small RNA libraries, total RNA was extracted from the pear tissue samples treated for 1 and 5 days at 24 °C and 37 °C using TRIzol reagent (Invitrogen, USA) according to the manufacturer’s instructions. Mixed samples containing equal amounts of total RNA from the 1- and 5-day treatments at 37 °C, were designated as T37, and a mixed sample containing equal amounts of the two treatments at 24 °C was the T24 control. The integrity and concentration of the total RNA samples were checked using a NanoDrop spectrophotometer and Agilent 2100 Bioanalyzer. High quality samples were used to construct small RNA library for sequencing on the Illumina Hiseq 2000 instrument at Beijing Genomics Institute (BGI) company (Shenzhen, China).

### Deep sequencing data analyses

All raw sequencing data was processed using the SOAP software (BGI Company) to obtain clean reads from each library as follows: except for low quality reads, reads with 5′ primer contaminants, reads without a 3′ primer, reads with no insert tags, reads with poly A tags, reads shorter than 18 nt and longer than 30 nt. Firstly, the sequences homologous to non-coding RNAs, including rRNAs, tRNAs, snRNAs, snoRNAs and siRNA were removed from the matched sequences through BLASTn searches using the NCBI Genebank (http://www.ncbi.nlm.nih.gov/blast/Blast.cgi/) and Rfam databases (http://www.sanger.ac.uk/Software/Rfam/). The remaining sequences were aligned to the pear genome sequence (http://peargenome.njau.edu.cn) with known miRNAs from miRBase17 and those matched to the pear genome shotgun-sequence assemblies were kept for known miRNA identification. Mireap software developed by BGI to predict novel miRNAs based on certain rules for stem-loop hairpins described [36–39], can be accessed from the following link: http://sourceforge.net/projects/mireap/.

### Differential expression analysis of miRNAs in response to high temperature

The expression levels of miRNAs in the 24 °C (control) and 37 °C (high temperature) treatments from in vitro-grown pear shoots were visualized by plotting the Log2-ratio of T37/T24. The procedures used were as follows: (1) The expression of miRNAs in the 24 °C and 37 °C libraries to get the number of transcripts per million (TPM) were normalized. Normalized expression = actual miRNA count/Total number of clean reads*1000000. In addition, the normalized expression of the miRNA was corrected to 0.01, when the miRNA gene count was zero. (2) The fold-change and *P-value* from the normalized expression were calculated. Fold-change = log_2_(T37 treatment/T24 control); the *P-value* was calculated as reported previously [60, 61]. The expression levels of miRNAs with fold changes >1.0-fold and *P -values* < 0.05 were considered to be responsive to high temperature treatment in *P. pyrifolia* shoots.

### Prediction of potential target genes for miRNA candidates in *P. pyrifolia*

Differentially expressed miRNAs were used as query sequences in BLASTn searches against the pear unigene database (http://peargenome.njau.edu.cn). The criteria for predicted mRNA target genes by alignment with each miRNA were based on those previously reported [62, 63].

### Real-time PCR analysis of miRNA and target gene expression

Real-time PCR was used to confirm the miRNA expression levels obtained from the high-throughput sRNA sequencing. Total RNAs were extracted from the samples treated for 1 and 5 days at 24 °C and 37 °C, respectively, using CTAB methods as described [64]. For miRNA, poly (A) tails were first added to the 3′ ends, and cDNA was then synthesized by RT using the One Step Prime-Script miRNA cDNA Synthesis Kit with a special oligo-dT adaptor according to the manufacturer’s instructions (TaKaRa, Dalian, China). The cDNA products were used as templates for quantitative PCR analysis, which was performed on a CFX96 Real-time System (BIO-RAD, USA) using SYBR *Premix Ex Taq* II (TaKaRa). All specific miRNA forward primers were designed based on the mature miRNA sequence, while the reverse primers were the adaptor sequence provided in One Step Prime-Script miRNA cDNA Synthesis Kit (Additional file10). In addition, qRT-PCR was used to analyze the expression of target genes cleaved by miRNAs and ASGV *cp* mRNAs, and the primers are also listed in Additional file 10. Total RNA digested by DNAase as template was reverse-transcribed using M-MLV reverse transcriptase (Promega, USA) and 6-base random primers to obtain cDNA. Each RT reaction contained 2.5 μl diluted cDNA, 12.5 μl of the SYBR *Premix Ex Taq* II (2×) PCR mixture, 1 μl of each 5 mM primer, and sterile water to a final volume 25 μl. The *Actin* gene was used as the internal reference gene. The specificity of the primers was verified by analysis of the melting curves from a thermal denaturing cycle of 60–95 °C with 1 °C increments applied for 1 s. All reactions were run in triplicate. The real-time PCR program conditions were as the follows: 95 °C for 30s, followed by 40 cycles of 95 °C for 5 s, and 60 °C for 30 s. The values for each miRNA/mRNA in the T24 sample were set as 1, and miRNA/mRNA relative expression level changes were calculated by a comparative *C*_T_ method (ΔΔC_T_) using the formula 2^-ΔΔCt^ [65].

## Additional files

Additional file 1: Table S1. Summary of clean read data produced by small RNA sequencing of the libraries constructed from the 24 °C and 37 °C treatments of in vitro-grown pear shoots. (DOC 32 kb) Additional file 2: Table S2. Summary of common and specific sRNA sequences and mean frequencies in the 24 °C and 37 °C libraries constructed from in vitro-grown pear shoots. (DOC 30 kb) Additional file 3: Table S3. Known miRNAs detected in the T24 and T37 libraries. (DOC 245 kb) Additional file 4: Table S4. Twelve differentially-expressed novel miRNAs and 20 equally-expressed novel miRNAs with miRNA*s in *P. pyrifolia*. (DOC 86 kb) Additional file 5: Figure S1. The precursors of 32 novel miRNAs and their hairpin structures in *P. pyrifolia*. The mature miRNAs are shown in yellow and miRNA*s underlined in green. The numbers show the base locations. (DOC 770 kb) Additional file 6:
**Detailed information about the loci, the corresponding precursor sequences and structures, and the alignments of all reads for each hairpin of the 32 potentially novel miRNAs and 109 candidate miRNAs.** (TXT 762 kb) Additional file 7: Table S5. The 109 candidate novel miRNAs detected in *P. pyrifolia*. (DOC 222 kb) Additional file 8: Figure S2. First nucleotide bias in novel miRNA candidates isolated from in vitro-grown pear shoots; (A) the 24 °C library, and (B) the 37 °C library. (DOC 66 kb) Additional file 9: Figure S3. Nucleotide bias at each position in novel miRNA candidates from in vitro-grown pear shoots at each position in (A) the 24 °C library, and (B) the 37 °C library. (DOC 61 kb) Additional file 10: Table S6. Oligonucleotide primers used for qRT-PCR expression analysis of miRNAs, mRNAs, and ASGV *cp* genes isolated from in vitro-grown shoots of *P. pyrifolia*. (DOC 50 kb)

## References

[1] Bartel DP (2004). MicroRNA: Genomics, biogenesis, mechanism, and function. Cell.

[2] Ding SW, Li H, Lu R, Li F, Li WX (2004). RNA silencing: a conserved antiviral immunity of plants and animals. Virus Res.

[3] Ding SW, Voinnet O (2007). Antiviral immunity directed by small RNAs. Cell.

[4] Arikit S, Zhai J, Meyers BC (2013). Biogenesis and function of rice small RNAs from non-coding RNA precursors. Curr Opin Plant Biol.

[5] Bouché N, Lauressergues D, Gasciolli V, Vaucheret H (2006). Anantagonistic function for Arabidopsis DCL2 in developmentand a newfunction for DCL4 in generating viral siRNAs. EMBO J.

[6] Jones-Rhoades MW, Bartel DP, Bartel B (2006). MicroRNAs and their regulatory roles in plants. Annu Rev Plant Biol.

[7] Vaucheret H (2006). Post-transcriptional small RNA pathways in plants: mechanism and regulations. Genes Dev.

[8] Chen X (2009). Small RNAs and their roles in plant development. Annu Rev Cell Dev Biol.

[9] Axtell MJ (2013). Classification and comparison of small RNAs from plants. Annu Rev Plant Biol.

[10] Rogers K, Chen X (2013). Biogenesis, turnover, and mode of action of plant microRNAs. Plant Cell.

[11] Guo C, Li L, Wang XF, Liang C (2015). Alterations in siRNA and miRNA expression profiles detected by deep sequencing of transgenic rice with siRNA-mediated viral resistance. PLoS One.

[12] Mlotshwa S, Pruss GL, Vance V (2008). Small RNAs in viral infection and host defense. Trends Plant Sci.

[13] Voinnet O (2009). Origin, biogenesis, and activity of plant microRNAs. Cell.

[14] Khraiwesh B, Zhu JK, Zhu J (1819). Role of miRNAs and siRNAs in biotic and abiotic stress responses of plants. Biochim Biophys Acta.

[15] Li F, Pignatta D, Bendix C, Brunkard JO, Cohn MM, Tung J (2012). MicroRNA regulation of plant innate immune receptors. Proc Natl Acad Sci U S A.

[16] Kumar R (2014). Roles of microRNA in biotic and abiotic stress responses in crop plants. Appl Biochem Biotechnol.

[17] Bazzini AA, Hopp HE, Beachy RN, Asurmendi S (2007). Infection and coaccumulation of tobacco mosaic virus proteins alter microRNA levels, correlating with symptoms and plant development. Proc Natl Acad Sci U S A.

[18] Naqvi AR, Haq QM, Mukherjee SK (2010). MicroRNA profiling of tomato leaf curl New Delhi virus (tolcndv) infected tomato leaves indicates that deregulation of mir159/319 and mir172 might be linked with leaf curl disease. Virol J.

[19] Chen H, Zhang LR, Yu KF, Wang AM (2015). Pathogenesis of *Soybean mosaic virus* in soybean carrying *Rsv1* gene is associated with miRNA and siRNA pathways, and breakdown of AGO1 homeostasis. Virology.

[20] Romanel E, Silva TF, Corrêa RL, Farinelli L, Hawkins JS, Schrago CEG (2012). Global alteration of microRNAs and transposon-derived small RNAs in cotton (*Gossypium hirsutum*) during Cotton leafroll dwarf polerovirus (CLRDV) infection. Plant Mol Biol.

[21] Vaucheret H, Mallory AC, Bartel DP (2006). AGO1 homeostasis entails coexpression of MIR168 and AGO1 and preferential stabilization of miR168 by AGO1. Mol Cell.

[22] Havelda Z, Várallyay É, Valoczi A, Burgyán J (2008). Plant virus infection-induced persistent host gene downregulation in systemically infected leaves. Plant J.

[23] Várallyay É, Válóczi A, Ágyi Á, Burgyán J, Havelda Z (2010). Plant virus-mediated induction of miR168 is associated with repression of ARGONAUTE1 accumulation. EMBO J.

[24] Várallyay É, Havelda Z (2013). Unrelated viral suppressors of RNA silencing mediate the control of ARGONAUTE1 level. Mol Plant Pathol.

[25] Mallory AC, Vaucheret H (2009). ARGONAUTE 1 homeostasis invokes the coordinate action of the microRNA and siRNA pathways. EMBO Rep.

[26] Wang GP, Hong N, Zhang ZP (1994). Identification of virus species in pears cultivated in northern China. China Fruits.

[27] Plese N, Hoxha E, Milicic D (1975). Pathological anatomy of trees affected with apple stem grooving virus. Phytopathology.

[28] Yanase H (1983). Back transmission of apple stem grooving virus to apple seedlings and induction of symptoms of apple topworking disease in Mitsuba Kaido (*Malus sieboldii*) and Kobano Zumi (*Malus sieboldii* var. arborescens) rootstocks. Acta Hortic.

[29] Tiziano C, Folwell RJ, Wandschneider P, Eastwell KC, Howell WE (2003). Economic implications of a virus prevention program in deciduous tree fruits in the US. Crop Prot.

[30] Wang QC, Cuellar WJ, Rajamaki ML, Hirata Y, Valkone JT (2008). Combined thermotherapy and cryotherapy for efficient virus eradication: Realation of virus distribution, subcellular changes, cell survival and viral RNA degradation in shoor tips. Mol Plant Pathol.

[31] Wang LP, Hong N, Wang GP, Xu WX, Michelutti R, Wang AM (2010). Distribution of apple stem grooving virus and apple chlorotic leaf spot virus in infected *in vitro* pear shoots. Crop Prot.

[32] Francki RIB, Fauquet CM, Knudson DL, Brown F (1991). Classification and nomenclature of viruses: fifth report of the international committee on taxonomy of viruses. Arch Virol.

[33] Wang T, Pan HT, Wang J, Yang WR, Cheng TR, Zhang QX (2014). Identification and profiling of novel and conserved microRNAs during the flower opening process in *Prunus mume* via deep sequencing. Mol Genet Genomics.

[34] Pantaleo V, Szittya G, Moxon S, Miozzi L, Moulton V, Dalmay T (2010). Identification of grapevine microRNAs and their targets using high throughput sequencing and degradome analysis. Plant J.

[35] Xia R, Zhu H, An Y, Beers EP, Liu Z (2012). Apple miRNAs and tasiRNAs with novel regulatory networks. Genome Biol.

[36] Hofacker IL, Fontana W, Stadler PF, Bonhoeffer LS, Tacker M, Schuster P (1994). Fast folding and comparison of RNA secondary structures. Monatsh Chem/Chem Mon.

[37] Bonnet E, Wuyts J, Rouzé P, Van de Peer Y (2004). Evidence that microRNA precursors, unlike other non-coding RNAs, have lower folding free energies than random sequences. Bioinformatics.

[38] Krol J, Sobczak K, Wilczynska U, Drath M, Jasinska A, Kaczynska D (2004). Structural features of microRNA (miRNA) precursors and their relevance to miRNA biogenesis and small interfering RNA/short hairpin RNA design. J Biol Chem.

[39] Kozomara A, Griffiths-Jones S (2014). miRBase: annotating high confidence microRNAs using deep sequencing data. Nucleic Acids Res.

[40] Wu J, Wang ZW, Shi ZB, Zhang S, Ming R, Zhu SL (2013). The genome of the pear (*Pyrus bretschneideri* Rehd.). Genome Res.

[41] Xin M, Wang Y, Yao Y, Xie C, Peng H, Ni Z (2010). Diverse set of microRNAs are responsive to powdery mildew infection and heat stress in wheat (*Triticum aestivum* L.). BMC Plant Biol.

[42] Gao ZH, Shi T, Luo XY, Zhang Z, Zhuang WB, Wang LJ (2012). High-throughput sequencing of small RNAs and analysis of differentially expressed microRNAs associated with pistil development in Japanese apricot. BMC Genomics.

[43] Sunkar R, Li YF, Jagadeeswaran G (2012). Functions of microRNAs in plant stress responses. Trends Plant Sci.

[44] Jagadeeswaran G, Saini A, Sunkar R (2009). Biotic and abiotic stress down-regulate miR398 expression in *Arabidopsis*. Planta.

[45] Feng JL, Liu X, Lai L, Chen JS (2011). Spatio-temporal expression of miRNAs in tomato tissues upon Cucumber mosaic virus and Tomato aspermy virus infections. Acta Biochim Biophys Sin.

[46] Abreu PMV, Gaspar CG, Buss DS, Ventura JA, Ferreira PCG, Fernandes PMB (2014). Carica papaya MicroRNAs are responsive to papaya meleira virus Infection. PLoS One.

[47] Ranocha P, Chabannes M, Chamayou S, Danoun S, Jauneau A, Boudet AM (2002). Laccase down-regulation causes alterations in phenolic metabolism and cell wall structure in *poplar*. Plant Physiol.

[48] Olsen AN, Ernst HA, Leggio LL, Skriver K (2005). NAC transcription factors: Structurally distinct, functionally diverse. Trends Plant Sci.

[49] Wang X, Goregaoker SP, Culver JN (2009). Interaction of the tobacco mosaic virus replicase protein with a NAC domain transcription factor is associated with the suppression of systemic host defense. J Virol.

[50] Nuruzzaman M, Sharoni AM, Kikuchi S (2013). Roles of NAC transcription factors in the regulation of biotic and abiotic stress responses in plants. Front Microbiol.

[51] Cillo F, Mascia T, Pasciuto MM, Gallitelli D (2009). Differential effects of mild and severe cucumber mosaic virus strains in the perturbation of microRNA-regulated gene expression in tomato map to the 30 sequence of RNA 2. Mol Plant Microbe Interact.

[52] Feng JL, Liu SS, Wang MN, Lang QL, Jin CZ (2014). Identification of microRNAs and their targets in tomato infected with cucumber mosaic virus based on deep sequencing. Planta.

[53] Aida M, Ishida T, Tasaka M (1999). Shoot apical meristem and cotyledon formation during *Arabidopsis* embryogenesis: interaction among the CUP-SHAPED COTYLEDON and shoot meristemless genes. Development.

[54] Takada S, Hibara K, Ishida T, Tasaka M (2001). The CUPSHAPED COTYLEDON1 gene of *Arabidopsis* regulates shoot apical meristem formation. Development.

[55] Heisler MG, Ohno C, Das P, Sieber P, Reddy GV, Long JA (2005). Patterns of auxin transport and gene expression during primordium development revealed by live imaging of the *Arabidopsis* inflorescence meristem. Curr Biol.

[56] Harrison BD (1956). Studies on the effect of temperature on virus multiplication in inoculated leaves. Annu Appl Biol.

[57] An FM, Hsiao SR, Chan MT (2011). Sequencing-based approaches reveal low ambient temperature-responsive and tissue-specific MicroRNAs in *Phalaenopsis Orchid*. PLoS One.

[58] Li MY, Wang F, Xu ZS, Jiang Q, Ma J, Tan GF (2014). High throughput sequencing of two celery varieties small RNAs identifies microRNAs involved in temperature stress response. BMC Genomics.

[59] Wang LP, Wang GP, Hong N, Tang RR, Deng XY (2006). Effect of thermotherapy on elimination of apple stem grooving virus and apple chlorotic leaf spot virus in tips of in vitro-cultured pear shoots. HortScience.

[60] Jiang P, Wu H, Wang W, Ma W, Sun X, Lu Z (2007). MiPred: classification of real and pseudo microRNA precursors using random forest prediction model with combined features. Nucleic Acids Res.

[61] Li G, Li Y, Li X, Ning X, Li M, Yang G (2011). MicroRNA identity and abundance in developing swine adipose tissue as determined by Solexa sequencing. J Cell Biochem.

[62] Allen E, Xie Z, Gustafson AM, Carrington JC (2005). MicroRNA-directed phasing during trans-acting siRNA biogenesis in plants. Cell.

[63] Schwab R, Palatnik JF, Riester M, Schommer C, Schmid M, Weigel D (2005). Specific effects of microRNAs on the plant transcriptome. Dev Cell.

[64] Angelini E, Clair D, Borgo M, Bertaccini A, Boudon-Padieu E (2001). *Flavescence dorée* in France and Italy. Occurrence of closely related phytoplasma isolates and their near relationships to palatinate grapevine yellows and an alder phytoplasma. Vitis.

[65] Livak KJ, Schmittgen TD (2001). Analysis of relative gene expression data using real-time quantitative PCR and the 2^-ΔΔCT^ method. Methods.

