# Supplementary material for: Identification and characterization of microRNAs from in vitro-grown pear shoots infected with Apple stem grooving virus in response to high temperature using small RNA sequencing
Source: BMC Genomics. 2015 Nov 16;16:945. doi: 10.1186/s12864-015-2126-8 (PMC4647338; doi:10.1186/s12864-015-2126-8)
Supplement: Additional file 1: Table S1. — Summary of clean read data produced by small RNA sequencing of the libraries constructed from the 24 °C and 37 °C treatments of in vitro-grown pear shoots. (DOC 32 kb) [file 12864_2015_2126_MOESM1_ESM.doc]

**Table S1** Summary of clean read data produced by small RNA sequencing of the libraries constructed from the 24°C and 37°C treatments of *in vitro*-grown pear shoot.

| Type | 24°C | | 37°C | |
| --- | --- | --- | --- | --- |
|  | Count | Percent (%) | Count | Percent (%) |
| Total reads | 22,741,370 |  | 20,540,362 |  |
| High quality | 22,688,052 | 100 | 20,494,256 | 100 |
| 3' adapter null | 59,140 | 0.26 | 38,599 | 0.19 |
| Insert null | 1,280 | 0.01 | 1,322 | 0.01 |
| 5' adapter contaminants | 21,715 | 0.10 | 18,380 | 0.09 |
| Smaller than 18 nt | 11,391 | 0.05 | 23,592 | 0.12 |
| Poly(A) | 1,529 | 0.01 | 1,109 | 0.01 |
| Clean reads | 22,592,997 | 99.58 | 20,411,254 | 99.59 |
