# Supplementary material for: Identification and characterization of microRNAs from in vitro-grown pear shoots infected with Apple stem grooving virus in response to high temperature using small RNA sequencing
Source: BMC Genomics. 2015 Nov 16;16:945. doi: 10.1186/s12864-015-2126-8 (PMC4647338; doi:10.1186/s12864-015-2126-8)
Supplement: Additional file 2: Table S2. — Summary of common and specific sRNA sequences and mean frequencies in the 24 °C and 37 °C libraries constructed from in vitro-grown pear shoots. (DOC 30 kb) [file 12864_2015_2126_MOESM2_ESM.doc]

**Table S2** Summary of common and specific sRNA sequences and mean frequencies in the 24°C and 37°C libraries constructed from *in vitro*-grown pear shoots.

| Class | Unique sRNAs | Percent (%) | Total sRNAs | Percent (%) | Mean frequency |
| --- | --- | --- | --- | --- | --- |
| Total_sRNAs | 8242995 | 100.00% | 43004251 | 100.00% | 5.22 |
| T24_&_T37 | 1260839 | 15.30% | 35026601 | 81.45% | 27.78 |
| T24_specific | 4268224 | 51.78% | 5008275 | 11.65% | 1.17 |
| T37_specific | 2713932 | 32.92% | 2969375 | 6.90% | 1.09 |
