# Supplementary material for: Identification and characterization of microRNAs from in vitro-grown pear shoots infected with Apple stem grooving virus in response to high temperature using small RNA sequencing
Source: BMC Genomics. 2015 Nov 16;16:945. doi: 10.1186/s12864-015-2126-8 (PMC4647338; doi:10.1186/s12864-015-2126-8)
Supplement: Additional file 3: Table S3. — Known miRNAs detected in the T24 and T37 libraries. (DOC 245 kb) [file 12864_2015_2126_MOESM3_ESM.doc]

**Table S3** Known miRNAs detected in the T24 and T37 libraries.

| **Family** | **miRNA name** | **T24** | | **T37** | |
| --- | --- | --- | --- | --- | --- |
|  |  | **Counts** | **Normalised** | **Counts** | **Normalised** |
| miR156 | miR156b | 3262072 | 144384.2 | 2640071 | 129343.9 |
|  | miR156g-3p | 495 | 21.9094 | 339 | 16.6085 |
|  | miR157a | 4929222 | 218174.8 | 7049057 | 345351.5 |
|  | miR157d-3p | 381 | 16.8636 | 460 | 22.5366 |
| miR158 | miR158b | 154 | 6.8163 | 102 | 4.9972 |
|  | miR158b-3p | 765 | 33.86 | 789 | 38.6551 |
| miR159 | miR159-3p | 2248 | 99.4999 | 2082 | 102.0026 |
|  | miR159a | 1550 | 68.6053 | 1619 | 79.319 |
|  | miR319a-3p | 100 | 4.4262 | 116 | 5.6831 |
|  | miR319c | 296 | 13.1014 | 340 | 16.6575 |
| miR160 | miR160a | 308 | 13.6325 | 4592 | 11.3663 |
|  | miR160b-3p | 1960 | 86.7525 | 9082 | 77.5063 |
| miR162 | miR162a | 166 | 7.3474 | 161 | 7.8878 |
| miR164 | miR164a | 1854 | 82.0608 | 1706 | 83.5813 |
|  | miR164g-3p | 4 | 0.177 | 9 | 0.4409 |
| miR165 | miR165a | 865 | 38.2862 | 661 | 32.3841 |
|  | miR165a-3p | 386 | 17.0849 | 384 | 18.8132 |
| miR166 | miR166aw | 229972 | 10178.91 | 178336 | 8737.775 |
|  | miR166e-3p | 227493 | 10069.18 | 171146 | 8384.884 |
| miR167 | miR167a | 153045 | 6774.002 | 124749 | 6111.775 |
|  | miR167f-3p | 85 | 3.7622 | 137 | 6.712 |
| miR168 | miR168a | 14584 | 645.5098 | 9434 | 462.196 |
|  | miR168a-3p | 194 | 8.5867 | 196 | 9.6025 |
| miR169 | miR169g | 765 | 33.86 | 1007 | 49.3355 |
|  | miR169u-3p | 187 | 8.2769 | 176 | 8.6227 |
| miR171 | miR171 | 680 | 30.0978 | 705 | 34.5398 |
|  | miR171b-3p | 37 | 1.6377 | 46 | 2.2537 |
| miR172 | miR172a | 1905 | 84.3182 | 1816 | 88.9705 |
| miR390 | miR390a | 1993 | 88.2132 | 1251 | 61.2897 |
|  | miR390a-3p | 34 | 1.5049 | 23 | 1.1268 |
| miR391 | miR391 | 762 | 33.7273 | 640 | 31.3553 |
| miR393 | miR393a-3p | 30 | 1.3278 | 37 | 1.8127 |
|  | miR393d | 14 | 0.6197 | 10 | 0.4899 |
| miR394 | miR394a | 15 | 0.6639 | 23 | 1.1268 |
|  | miR394a-3p | 18 | 0.7967 | 12 | 0.5879 |
| miR395 | miR395a | 25 | 1.1065 | 24 | 1.1758 |
| miR396 | miR396b | 147 | 6.5064 | 184 | 9.0146 |
|  | miR396b-3p | 2440 | 107.9981 | 3438 | 168.4365 |
| miR397 | miR397a | 56596 | 2503.829 | 104360 | 5112.866 |
| miR398 | miR398b | 3198 | 141.5483 | 3402 | 166.6728 |
|  | miR398b-3p | 1786 | 79.051 | 2037 | 99.7979 |
| miR399 | miR399e | 2251 | 99.6326 | 2046 | 100.2388 |
| miR403 | miR403 | 337 | 14.9161 | 346 | 16.9514 |
| miR408 | miR408b | 333855 | 14776.92 | 555677 | 27224.05 |
| miR477 | miR477b | 38 | 1.6819 | 13 | 0.6369 |
|  | miR477b-3p | 43 | 1.9032 | 26 | 1.2738 |
| miR482 | miR482a-3p | 2147 | 95.0294 | 3181 | 155.8454 |
|  | miR482a-5p | 73992 | 3274.997 | 68366 | 3349.427 |
| miR529 | miR529 | 6567 | 290.6653 | 7792 | 381.7502 |
| miR530 | miR530a | 88 | 3.895 | 67 | 3.2825 |
| miR535 | miR535d | 438839 | 19423.6736 | 438839 | 20254.5615 |
| miR536 | miR536 | 354 | 15.6686 | 354 | 20.4789 |
| miR822 | miR822 | 328 | 14.5178 | 290 | 14.2078 |
| miR827 | miR827 | 1374 | 60.8153 | 1031 | 50.5114 |
| miR894 | miR894 | 7769 | 343.8676 | 9008 | 441.3252 |
| miR1026a | miR1026a | 26 | 1.1508 | 26 | 1.1268 |
| miR1043-3p | miR1043-3p | 43 | 1.9032 | 43 | 0.7839 |
| miR1310 | miR1310 | 269 | 11.9063 | 269 | 15.8246 |
| miR1432 | miR1432 | 545 | 24.1225 | 545 | 15.5796 |
| miR1511 | miR1511 | 2456 | 108.7063 | 2456 | 89.9994 |
| miR1520j | miR1520j | 57 | 2.5229 | 57 | 1.4208 |
| miR1526 | miR1526 | 835 | 36.9584 | 835 | 34.1968 |
| miR1854 | miR1854-5p | 90 | 3.9835 | 53 | 2.5966 |
| miR1863 | miR1863 | 63 | 2.7885 | 44 | 2.1557 |
| miR2111 | miR2111a-5p | 24 | 1.0623 | 21 | 1.0288 |
| miR2118 | miR2118a | 5157 | 228.2566 | 5949 | 291.4569 |
| miR2199 | miR2199 | 8103 | 358.651 | 11225 | 549.9417 |
| miR2592 | miR2592bl-5p | 12 | 0.5311 | 7 | 0.3429 |
| miR2867 | miR2867-3p | 1302 | 57.6285 | 949 | 46.494 |
| miR2873 | miR2873b | 456 | 20.1832 | 270 | 13.228 |
| miR2916 | miR2916 | 2663 | 117.8684 | 3886 | 190.3852 |
| miR2919 | miR2919 | 50 | 2.2131 | 28 | 1.3718 |
| miR3627 | miR3627-5p | 368 | 16.2882 | 1827 | 89.5094 |
| miR3699 | miR3699 | 126 | 5.5769 | 110 | 5.3892 |
| miR3711 | miR3711 | 27 | 1.1951 | 31 | 1.5188 |
| miR4234 | miR4234 | 44 | 1.9475 | 44 | 2.1557 |
| miR4369 | miR4369 | 255 | 11.2867 | 149 | 7.2999 |
| miR4371 | miR4371a | 44 | 1.9475 | 27 | 1.3228 |
| miR4382 | miR4382 | 46 | 2.036 | 53 | 2.5966 |
| miR4414 | miR4414a-5p | 5430 | 240.34 | 3822 | 187.2496 |
| miR4993 | miR4993 | 1677 | 74.2265 | 749 | 36.6954 |
| miR5021 | miR5021 | 180 | 7.9671 | 105 | 5.1442 |
| miR5029 | miR5029 | 601 | 26.6012 | 530 | 25.9661 |
| miR5059 | miR5059 | 793 | 35.0994 | 657 | 32.1881 |
| miR5072 | miR5072 | 211 | 9.3392 | 270 | 13.228 |
| miR5077 | miR5077 | 1298 | 57.4514 | 1508 | 73.8808 |
| miR5083 | miR5083 | 28 | 1.2393 | 44 | 2.1557 |
| miR5139 | miR5139 | 1659 | 73.4298 | 1212 | 59.379 |
| miR5183 | miR5183 | 72 | 3.1868 | 59 | 2.8906 |
| miR5213 | miR5213-3p | 73 | 3.2311 | 89 | 4.3603 |
| miR5217 | miR5217 | 309 | 13.6768 | 210 | 10.2884 |
| miR5225 | miR5225-3p | 8 | 0.3541 | 5 | 0.245 |
| miR5225 | miR5225c | 177 | 7.8343 | 181 | 8.8677 |
| miR5227 | miR5227 | 64 | 2.8327 | 42 | 2.0577 |
| miR5243 | miR5243 | 89 | 3.9393 | 60 | 2.9396 |
| miR5256 | miR5256 | 398 | 17.6161 | 300 | 14.6978 |
| miR5287 | miR5287b | 66 | 2.9213 | 40 | 1.9597 |
| miR5291 | miR5291a | 358 | 15.8456 | 256 | 12.5421 |
| miR5304 | miR5304-3p | 722 | 31.9568 | 413 | 20.2339 |
| miR5368 | miR5368 | 195 | 8.631 | 197 | 9.6515 |
| miR5386 | miR5386 | 2015 | 89.1869 | 1605 | 78.6331 |
| miR5485 | miR5485 | 12622 | 558.6687 | 15376 | 753.3099 |
| miR5519 | miR5519 | 18 | 0.7967 | 34 | 1.6657 |
| miR5537 | miR5537 | 14 | 0.6197 | 6 | 0.294 |
| miR5671 | miR5671 | 1756 | 77.7232 | 3110 | 152.3669 |
| miR5720 | miR5720 | 66 | 2.9213 | 70 | 3.4295 |
| miR5741 | miR5741a | 657 | 29.0798 | 421 | 20.6259 |
| miR5742 | miR5742 | 530 | 23.4586 | 291 | 14.2568 |
| miR5745b | miR5745b | 28 | 1.2393 | 45 | 2.2047 |
| miR5778 | miR5778 | 34 | 1.5049 | 20 | 0.9799 |
| miR5799 | miR5799 | 38 | 1.6819 | 27 | 1.3228 |
| miR5801 | miR5801 | 90 | 3.9835 | 40 | 1.9597 |
| miR5813 | miR5813 | 4098 | 181.3836 | 6201 | 303.803 |
| miR5830 | miR5830 | 41 | 1.8147 | 49 | 2.4006 |
| miR5837.1 | miR5837.1 | 464 | 20.5373 | 338 | 16.5595 |
| miR6107 | miR6107 | 6012 | 266.1002 | 2920 | 143.0583 |
| miR6151a | miR6151a | 441 | 19.5193 | 385 | 18.8621 |
| miR6171 | miR6171 | 556 | 24.6094 | 526 | 25.7701 |
| miR6173 | miR6173 | 55 | 2.4344 | 73 | 3.5765 |
| miR6214 | miR6214 | 2863 | 126.7207 | 3940 | 193.0308 |
| miR6267a | miR6267a | 199 | 8.808 | 149 | 7.2999 |
| miR6284 | miR6284 | 9 | 0.3984 | 14 | 0.6859 |
| miR6292 | miR6292 | 3177 | 140.6188 | 2039 | 99.8959 |
| miR6300 | miR6300 | 791 | 35.0108 | 1258 | 61.6327 |
| miR6460 | miR6460 | 14 | 0.6197 | 16 | 0.7839 |
| miR6466-5p | miR6466-5p | 13 | 0.5754 | 9 | 0.4409 |
| miR6475 | miR6475 | 26 | 1.1508 | 29 | 1.4208 |
| miR6478 | miR6478 | 176 | 7.79 | 248 | 12.1502 |
| miR6483 | miR6483 | 43 | 1.9032 | 27 | 1.3228 |
| miR6485 | miR6485 | 41 | 1.8147 | 51 | 2.4986 |
| miR7120a | miR7120a | 4888 | 216.3502 | 3616 | 177.1572 |
| miR7121d | miR7121d | 30729 | 1360.1117 | 27937 | 1368.7057 |
| miR7122a | miR7122a | 5407 | 239.3219 | 6946 | 340.3025 |
| miR7123a | miR7123a | 753 | 33.3289 | 351 | 17.1964 |
| miR7124a | miR7124a | 152 | 6.7277 | 152 | 7.4469 |
| miR7125 | miR7125 | 252 | 11.1539 | 378 | 18.5192 |
| miR7126 | miR7126 | 330 | 14.6063 | 284 | 13.9139 |
| miR7532a | miR7532a | 5014 | 221.9272 | 4729 | 231.6859 |
| miR7736-5p | miR7736-5p | 208 | 9.2064 | 143 | 7.0059 |
| miR7753-3p | miR7753-3p | 382 | 16.9079 | 445 | 21.8017 |
| miR7782-3p | miR7782-3p | 1495 | 66.1709 | 876 | 42.9175 |
| miR7817b | miR7817b | 270 | 11.9506 | 283 | 13.8649 |
| miR7822 | miR7822 | 12999 | 575.3553 | 7970 | 390.4709 |
| miR8016 | miR8016 | 55 | 2.4344 | 44 | 2.1557 |
| miR8051-5p | miR8051-5p | 1529 | 67.6758 | 1221 | 59.8199 |
| miR8126-5p | miR8126-5p | 224 | 9.9146 | 120 | 5.8791 |
| miR8133-5p | miR8133-5p | 145 | 6.4179 | 109 | 5.3402 |
| miR8136 | miR8136 | 46 | 2.036 | 37 | 1.8127 |
| miR8155 | miR8155 | 114 | 5.0458 | 75 | 3.6744 |
