# Supplementary material for: Identification and characterization of microRNAs from in vitro-grown pear shoots infected with Apple stem grooving virus in response to high temperature using small RNA sequencing
Source: BMC Genomics. 2015 Nov 16;16:945. doi: 10.1186/s12864-015-2126-8 (PMC4647338; doi:10.1186/s12864-015-2126-8)
Supplement: Additional file 4: Table S4. — Twelve differentially-expressed novel miRNAs and 20 equally-expressed novel miRNAs with miRNA*s in P. pyrifolia. (DOC 86 kb) [file 12864_2015_2126_MOESM4_ESM.doc]

**Table S4** Twelve differentially-expressed novel miRNAs and 20 equally-expressed novel miRNAs with miRNA*s in *P. pyrifolia*.

| miRNA name | Length of  mature(nt) | Mature sequence( 5′→3′) | Counts of miRNAs/miRNA*s | Precursor location | Length of precursor(nt) | Mature sequence locus (Arm ) | MFE  (kcal/mol) |
| --- | --- | --- | --- | --- | --- | --- | --- |
| novel160 | 21 | UUGAGCCGUGCCAAUAUCACG | 86/14 | scaffold27.0:729747:729852:- | 106 | 3p | -46.40 |
| novel197 | 23 | CUCUUGACCGUUAGAUUUGGCUU | 37/10 | scaffold322.0:453344:453487:+ | 144 | 3p | -39.20 |
| novel241 | 21 | UGGUGCAGGUCGGGAACCGCU | 145/119 | scaffold427.0:352617:352765:+ | 149 | 5p | -73.30 |
|  | 21 | UGGUGCAGGUCGGGAACCGCU | 145/119 | scaffold494.0:207339:207487:+ | 149 | 5p | -73.30 |
| novel262 | 21 | AGUGGAAGGGUAGGAAAGAAG | 18817/332 | scaffold48.0:691765:691886:- | 122 | 5p | -48.10 |
| novel290 | 22 | UUCAGCUUCCACUUCUUUUGGU | 57/31 | scaffold58.0:972403:972504:+ | 102 | 3p | -49.80 |
|  | 22 | UUCAGCUUCCACUUCUUUUGGU | 57/31 | scaffold58.0:986368:986469:+ | 102 | 3p | -49.80 |
| novel345 | 20 | UUGCAUAUCUCAGGAGCUGC | 961/13 | scaffold82.0:571491:571637:- | 147 | 3p | -53.50 |
| novel497 | 21 | CGAACUUGGUGGAUUAGGAGG | 22/1 | scaffold30.0:39482:39597:+ | 116 | 3p | -65.77 |
| novel540 | 21 | CGGGUCCCGCCUUGCAUCAAC | 216/153 | scaffold427.0:352617:352765:+ | 149 | 3p | -73.30 |
|  | 21 | CGGGUCCCGCCUUGCAUCAAC | 216/153 | scaffold494.0:207339:207487:+ | 149 | 3p | -73.30 |
| novel558 | 23 | UAACCGUUAGAUCUUUGUUUGAA | 22/5 | scaffold51.0:1029861:1029943:+ | 83 | 5p | -28.60 |
| novel564 | 21 | UUAGAUCAAGUUUGAACGGCC | 24/1 | scaffold551.0:29882:29991:- | 110 | 3p | -46.10 |
| novel566 | 23 | AGGUGAGACGUAUCCCGAGGACA | 30/1 | scaffold566.0:149932:150152:+ | 221 | 3p | -61.00 |
| novel615 | 22 | CAUAUCAUCUCUGAAAACGGCA | 118/1 | scaffold88.0:196339:196591:+ | 253 | 3p | -57.10 |
| novel1 | 21 | UUGGAUUAAAAUUGAACGGCC | 5530/25 | scaffold1.0:124796:124905:+ | 110 | 3p | -46.50 |
|  | 21 | UUGGAUUAAAAUUGAACGGCC | 5674/23 | scaffold404.0:200279:200388:+ | 110 | 3p | -45.90 |
|  | 21 | UUGGAUUAAAAUUGAACGGCC | 5551/25 | scaffold408.0:138866:138975:- | 110 | 3p | -47.40 |
|  | 21 | UUGGAUUAAAAUUGAACGGCC | 5551/23 | scaffold414.0:82703:82812:- | 110 | 3p | -41.80 |
|  | 21 | UUGGAUUAAAAUUGAACGGCC | 5530/25 | scaffold50.0:857101:857210:- | 110 | 3p | -45.70 |
| novel157 | 21 | UUGAGGAUGCAUAGUUUUCAG | 53870/25 | scaffold266.0:262299:262521:+ | 223 | 3p | -102.10 |
|  | 21 | UUGAGGAUGCAUAGUUUUCAG | 53870/25 | scaffold266.0:383731:383953:- | 223 | 3p | -102.10 |
| novel16 | 21 | UGUGAAUGAUGCGGGAGAUAU | 222/36 | scaffold103.0:770828:771097:- | 270 | 3p | -79.30 |
| novel169 | 23 | UAGGAUAGGAUAAGAUAAGGUUA | 112/1 | scaffold28.0:521136:521245:+ | 110 | 5p | -32.70 |
| novel179 | 21 | CAGCCAAGGAUGACUUGCCGG | 448/20 | scaffold292.0:202953:203207:+ | 255 | 5p | -99.40 |
|  | 21 | CAGCCAAGGAUGACUUGCCGG | 521/15 | scaffold292.0:202953:203206:+ | 254 | 5p | -98.90 |
| novel187 | 22 | UCCCCGGCAACGGUGCCAAAAA | 253/20 | scaffold3.0:1347347:1347448:+ | 102 | 3p | -63.66 |
| novel2 | 21 | UAACGAACUUGUCGUAGGAUG | 6915/501 | scaffold1.0:2288735:2288874:+ | 140 | 3p | -47.60 |
|  | 21 | UAACGAACUUGUCGUAGGAUG | 4913/497 | scaffold1.0:2288737:2288872:+ | 136 | 3p | -47.60 |
| novel227 | 21 | UCCAAGUUGAAGGGUACUCAU | 28/1 | scaffold393.0:361944:362077:+ | 134 | 5p | -52.53 |
| novel230 | 21 | UGAAGAUGACAAACUCUAUCU | 121/1 | scaffold40.0:732166:732293:+ | 128 | 3p | -63.10 |
| novel261 | 21 | AAUGGAAGGGUAGGAAAGAAG | 7185/184 | scaffold48.0:686769:686890:- | 122 | 5p | -50.00 |
| novel289 | 21 | UCUGAAAAUCAGAUCUGACAA | 202/3 | scaffold570.0:250121:250397:+ | 277 | 5p | -161.40 |
| novel292 | 22 | UCUUGAAUGAUCUAUCUUCGGU | 47/14 | scaffold588.0:45671:46019:- | 349 | 3p | -119.52 |
| novel3 | 21 | UGCCAAAGGAGAAUUGCCCUG | 61/3 | scaffold1.0:3610315:3610435:+ | 121 | 3p | -58.50 |
| novel349 | 21 | UGGUGUUUGGAUGGACGUGUU | 38511/440 | scaffold862.0:54349:54538:- | 190 | 5p | -84.50 |
| novel37 | 22 | ACAGUUGAAGCUGCCAGCAUGA | 358/2 | scaffold120.0:419196:419295:- | 100 | 5p | -47.10 |
| novel370 | 21 | UGAGCCAAGGAUGACUUGCCA | 613/3 | scaffold980.0:39076:39183:+ | 108 | 5p | -44.60 |
|  | 21 | UGAGCCAAGGAUGACUUGCCA | 613/3 | scaffold980.0:102616:102723:- | 108 | 5p | -44.60 |
| novel371 | 21 | GAGCCAAGGAUGAAUUGCCGG | 868/68 | scaffold980.0:132687:132813:+ | 127 | 5p | -47.10 |
|  | 21 | GAGCCAAGGAUGAAUUGCCGG | 868/68 | scaffold980.0:9256:9382:- | 127 | 5p | -47.10 |
| novel39 | 22 | UUUGAGUAUGAACAUGAGGAAC | 98/1 | scaffold1203.0:9350:9440:- | 91 | 3p | -20.10 |
| novel43 | 22 | AGCUUUGGGAUGUUGGCGCGUU | 102/28 | scaffold1257.0:27692:27812:+ | 121 | 5p | -37.42 |
|  | 22 | AGCUUUGGGAUGUUGGCGCGUU | 102/28 | scaffold719.0:138893:139013:+ | 121 | 5p | -38.12 |
| novel87 | 21 | UUGGAGAGGCGGUACAAAUGU | 354/1 | scaffold160.1:102109:102191:- | 83 | 3p | -55.20 |
