# Supplementary material for: Identification and characterization of microRNAs from in vitro-grown pear shoots infected with Apple stem grooving virus in response to high temperature using small RNA sequencing
Source: BMC Genomics. 2015 Nov 16;16:945. doi: 10.1186/s12864-015-2126-8 (PMC4647338; doi:10.1186/s12864-015-2126-8)
Supplement: Additional file 5: Figure S1. — The precursors of 32 novel miRNAs and their hairpin structures in P. pyrifolia. The mature miRNAs are shown in yellow and miRNA*s underlined in green. The numbers show the base locations. (DOC 770 kb) [file 12864_2015_2126_MOESM5_ESM.doc]

**Figure S1** The precursors of 32 novel miRNAs and their hairpin structures in *P. pyrifolia*. The mature miRNAs are shown in yellow and miRNA*s underlined in green. The numbers show the base locations.

**novel160**


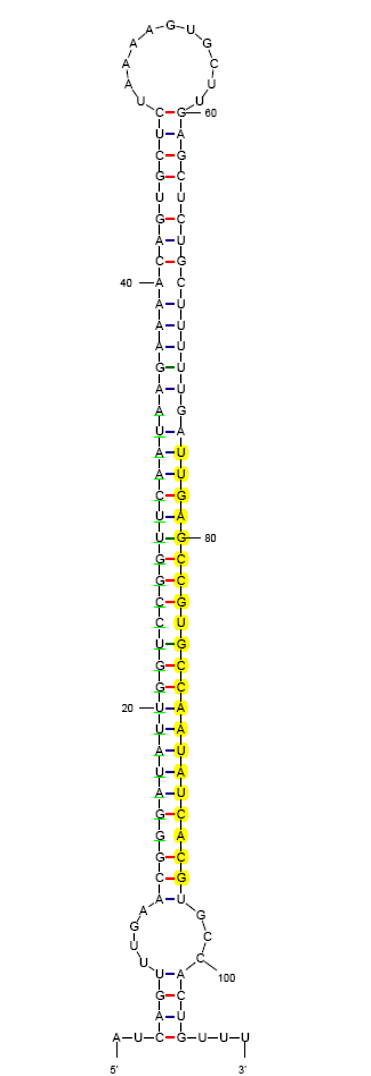
AUCAGUUUGAACGGGAUAUUGGUCCGGUUCAAUAAGAAAACAGUGCUCUAAAAGUGCUUGAGCUCUGCUUUUUGAUUGAGCCGUGCCAAUAUCACGUGCCACUGUUU

**novel197**

AUCCACAUUUAUCGGGUUUAACGGUUAGAAUUGCCAUCCCUAGUGUGUGUGUGUACGUAUAUAGGCCCUUUAACAAGAGGGAUCCCCAUUUUUCAAAAAGAUAGGGACAUUCUCUUGACCGUUAGAUUUGGCUUUAAUGAAAUU


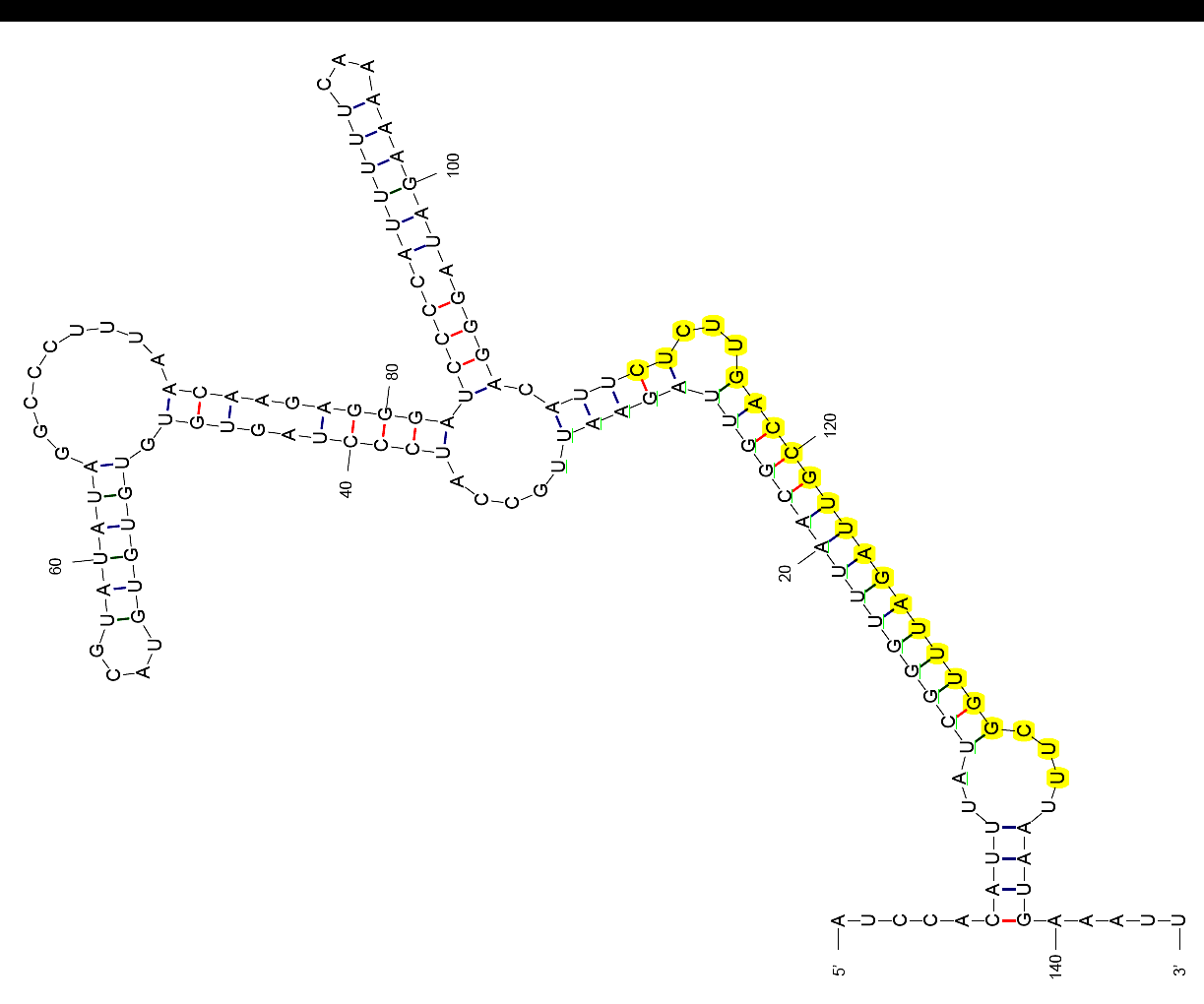


**novel241**

CUGGCGGUCUCUAAUUCGCUUGGUGCAGGUCGGGAACCGCUUUACCCUGCUUUGCCGUGUCUGGGCCUAUGAGCUAGGUGGCUCGAUGUAAUCGGUCACGGGGCAAAACGGGUCCCGCCUUGCAUCAACUGAAUCCGAGACCGCGGUGA


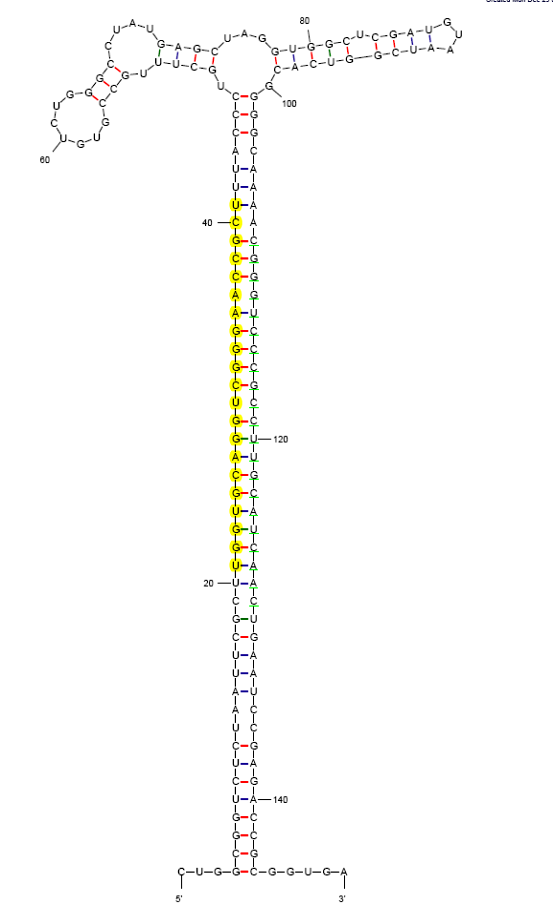


**novel262**

GGCUUUGAGGAGGUUUAUGGAGUGGAAGGGUAGGAAAGAAGCAACUUCUCUUUUUGGUCUCAAAUCCUAAAGAUCGAAUUUCUUUCCUAACCCUCCCAUUCCUUCAAUUUCCUAAAGUUUCU


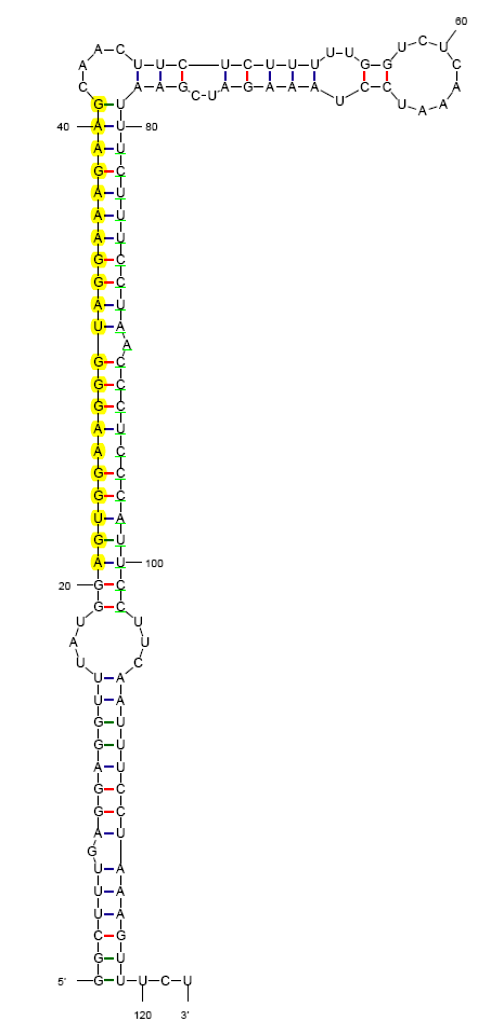


**novel290**

ACCUCUAAAAGAAGAUGCACCAGAGAAGUUGAAGUUGAAGAUGCACCAAAAGCUCUGAUCUUCAGCUUCCACUUCUUUUGGUGCAUCUUUUAUUAGACCUAU


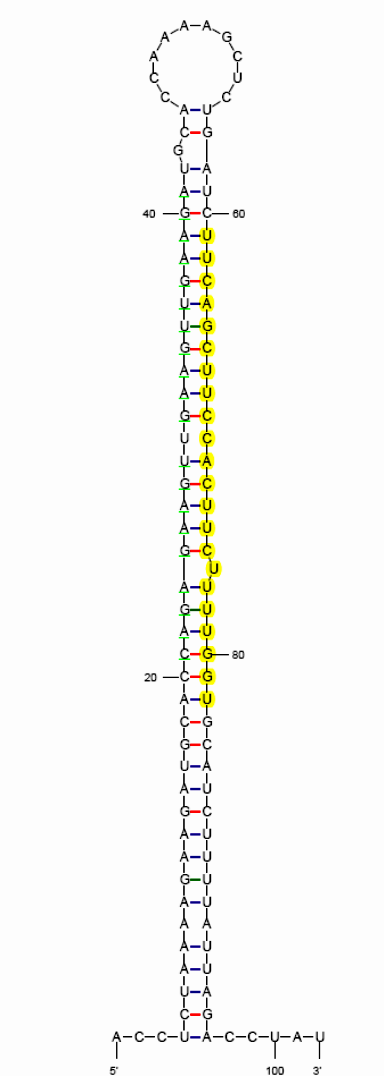


**novel345**

AGGUGAUGCUGGGUGGAUAGAGCUGCUGAGCUAUGGAUCCCAGAGUCCUAUAUGUCCUAUAUCUCAGAUCAGAUGAGAUGCGAUGGAAAGGAUGAUAGGCUUGUGGCUUGCAUAUCUCAGGAGCUGCAUUACCUGUACGCUGCCUCU

**novel497**

CGGAUCCUUUUCUCCUAAUUUAUCAAGUUCGGAGAUUCGGGCUAUUGAAUCAGUCGUUGAAUCAAAUUUCAGUAGCCUGGAUCCUCGAACUUGGUGGAUUAGGAGGAAAUUGAUCC


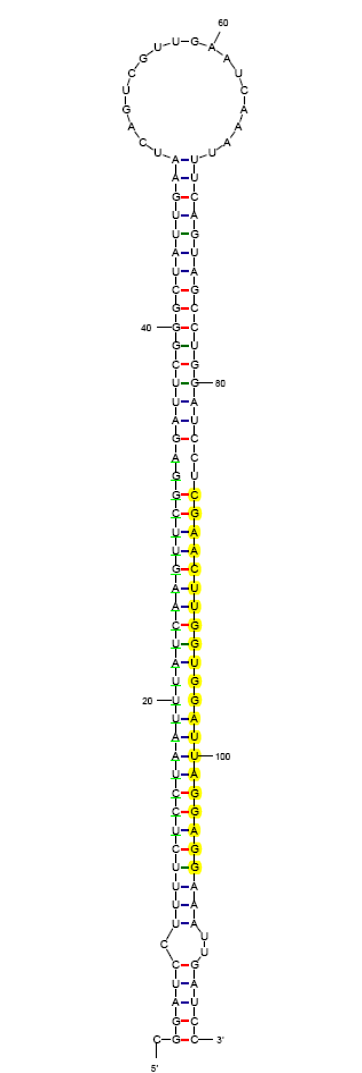


**novel540**

CUGGCGGUCUCUAAUUCGCUUGGUGCAGGUCGGGAACCGCUUUACCCUGCUUUGCCGUGUCUGGGCCUAUGAGCUAGGUGGCUCGAUGUAAUCGGUCACGGGGCAAAACGGGUCCCGCCUUGCAUCAACUGAAUCCGAGACCGCGGUGA


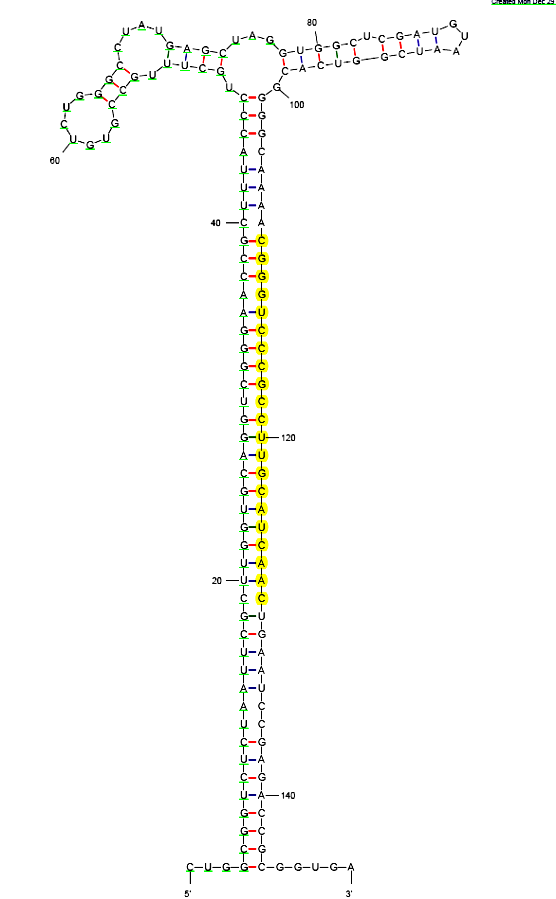


**novel558**

UCGGAUAUUUUAACCGUUAGAUCUUUGUUUGAAAAUACAAAAAUAAAGAUCUAAAAACGGUUAAAACACCUAAAGUAUCUGAU


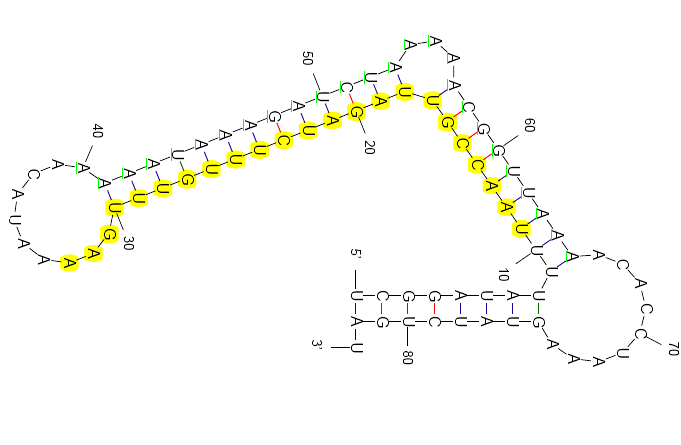


**novel564**

CAGCACAUCCGGGUCGUUGAAAUUUGAUCCAACGGCUCUAAAUAGGGAGCCCCUUUAAAAGUUAUAAUAAUUGUAAUCGUUAGAUCAAGUUUGAACGGCCCGAAUGGGCU

**novel615**

GUAAUGCACCUGCCACAUUUCAAAGAUGCAUGAUGAGCAUAUUUUCCGAUUAUGUUGAGAAAAUAAUUGAAGUUUUUAUGGAUGAUUUCAGUGUCUUUGGAGAUUCCUUUGAUGGUUGCUUGCAUAAUCUAAGUUUGAUUCUAAAACGUUGUGUUGAAACUAACCUUGUUCUUAAUUGGGAAAAAUGUCAUUUCAUGGUUAAACAAGGCAUAGUUUUAAGUCAUAUCAUCUCUGAAAACGGCAUUAAAGUUGA


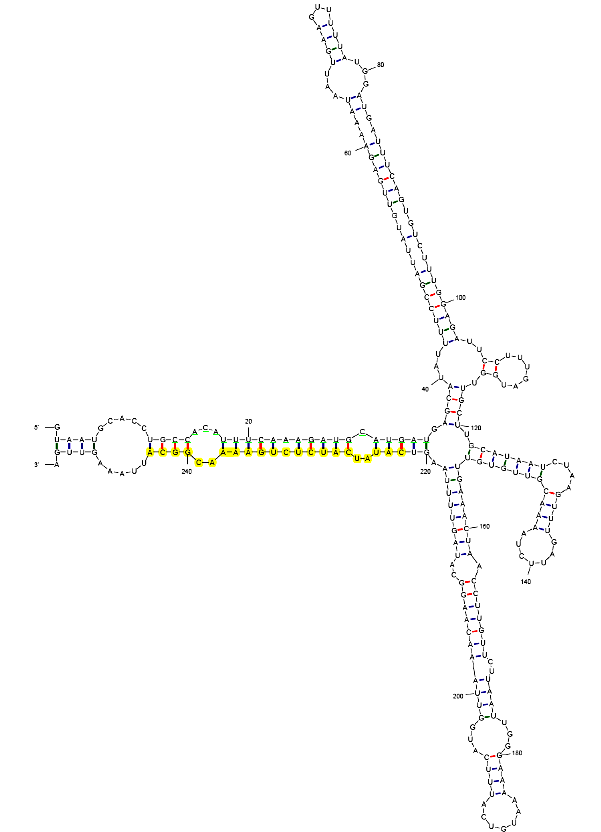


**novel1**

CACGAAAUCCAGACCGUUCAAUUUUAAUCCAACGGCUCUAAACAGAGGGUCUCCAAAAAGGUUAUAAUAAUUGUAACCGUUGGAUUAAAAUUGAACGGCCCGAAUUUCGU

**
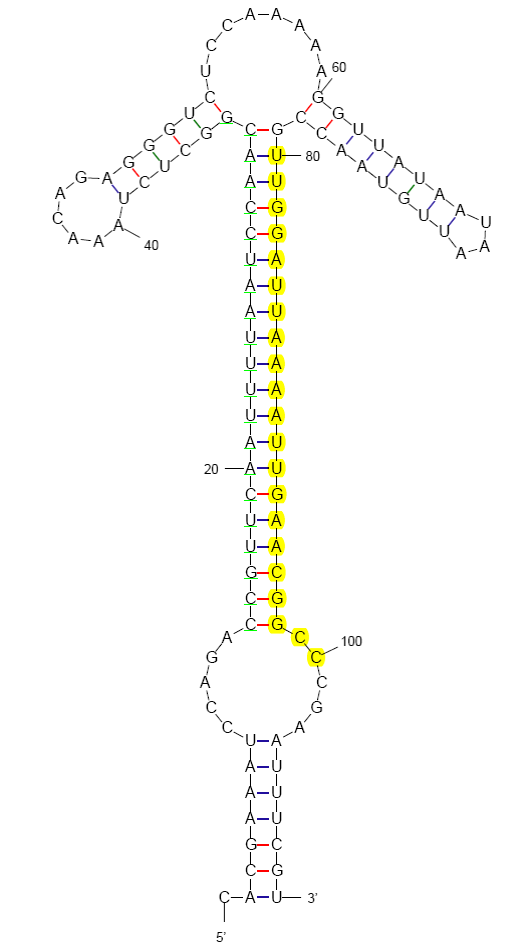
**

**novel157**

ACUGCAUUUUCCGAAAAUAAUGCAUCUUCAACGUGGUGUUUGGAUGAACGUGUUAAGAUCCUACAAAUGAAAAUAUUUCUACAAGGUGAAUCACUCGGAAGUACAAAAUCAGAGGAAAAUUUUUGGUACUUCUCAUACUUUUGAGGGGUUCAUCUUGUUGUAAGAUCUUAAUAAGUUCAUCCAAACACCAUAUUGAGGAUGCAUAGUUUUCAGAAAAUACAAU

**
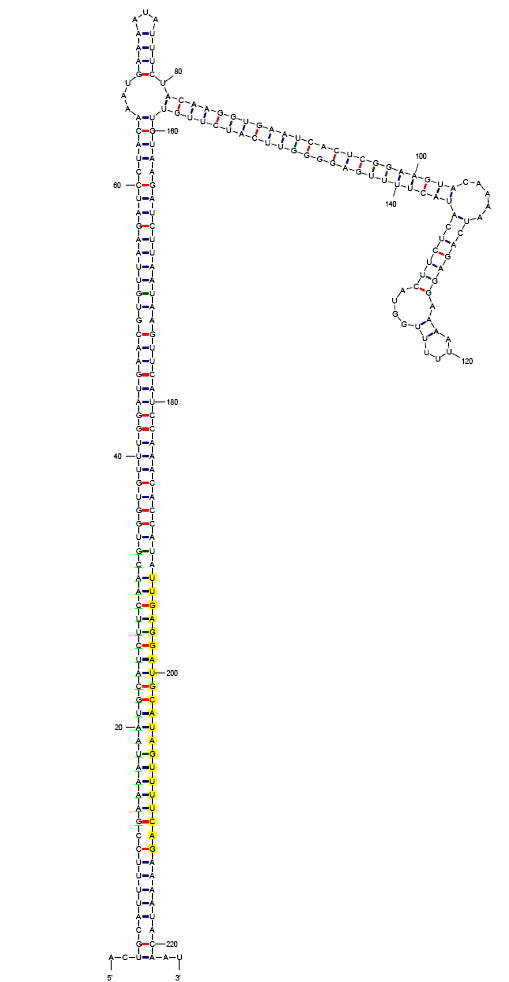
**

**novel16**


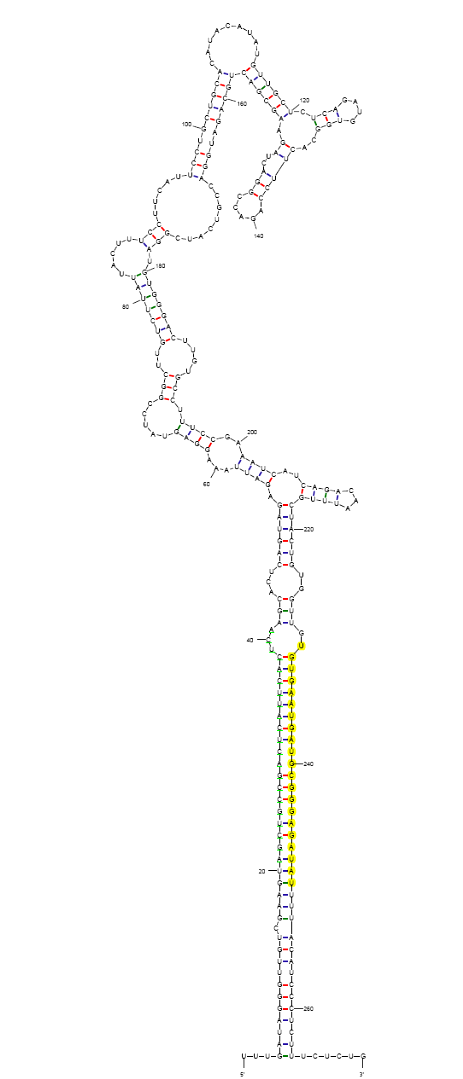
UUUGAUAGGGUUGUCGAAGUAGCUGCCGACUCAUUCACUCAAGCACUCAGUAGAGAUUAAAGGAGUAUCCGGCUUGUCUUAUUACUUUCCUUCAUUCCUGCUGCACAUACAUAUGUUGCUCUCAGAUGUGGCACUUCCAGACCGGACUAGAAGCGACUGCAGAUGGACCGUCAUCGGAUGUGGGACUUGUGCCUUUCCGAAAUCAUCAGACAAUUUGCUACUGUGGUUGUGUGAAUGAUGCGGGAGAUAUUUUACAUCCCUCUUUCUCUG

**novel169**

UAGGAAAAAUUAGGAUAGGAUAAGAUAAGGUUAGGUUGGAUAAGGUUUGGAUAAUGUUCUUUCUUUUUAGCUGAUUCCUUAUCUUCAAUGUCUUGAAUUAAUUCUUCCAG

**
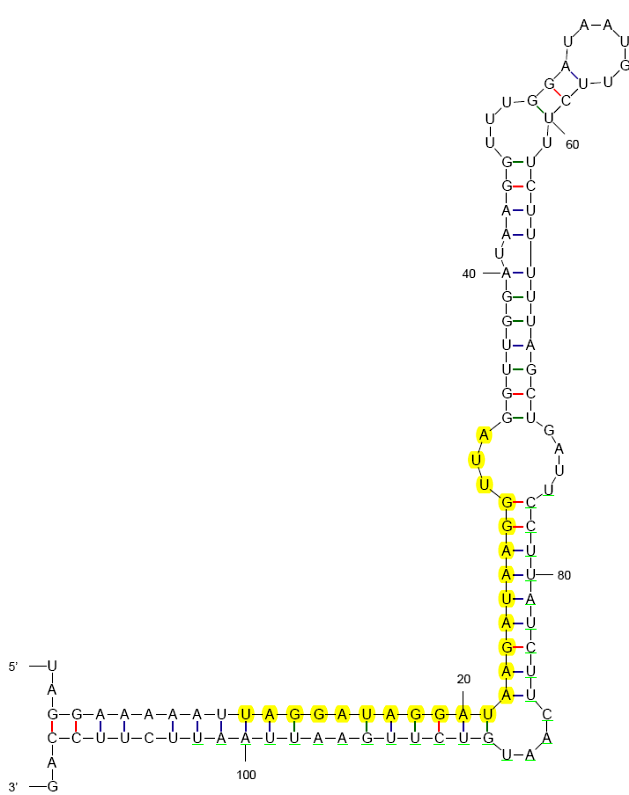
**

**novel179**

UGGGAACGAGGUAUGCAGUGCAGCCAAGGAUGACUUGCCGGCAACAAUGCUCCCAUACAAUAUUUGUAUAUCAACCCUAAUCUUGCAGAUCUAUAUAUGUAUAUAUGUAUAUGUGCAUAGCAUAUACAUAUGGGAUGUAUCAUGUAUGUUGAUGUCAUCUCAUGCGUAUAUGCAUGAUAGAUAAGAUACAUGAUCUUGUGACAAUGAGUUGAUCGGCAAGUCGUCUCUGGCUACAUUUUUGCCUCCUUUUCUCAU

**novel_mir_187**

**novel187**

CGUUCUGCAAUUUUUGGUACCGUUUCGGGAAGCAGCUUGAGAAAUACUCUAGCGCUCCUUCUCAAGCCGCUCCCCGGCAACGGUGCCAAAAAUUGCAGAACC

**novel2**

GUAUUCAAUUCAUCCUCCAACAAGUCCGUGAUCUCUAUGCGCAAUCUCUUAAUUUAUGAUAAAAUCAACAAAGAAAUGCUAUGAAAAGAAAGUAGUUUGCGGAUAGAGAUAACGAACUUGUCGUAGGAUGAGCUGAAGAU

**novel227**


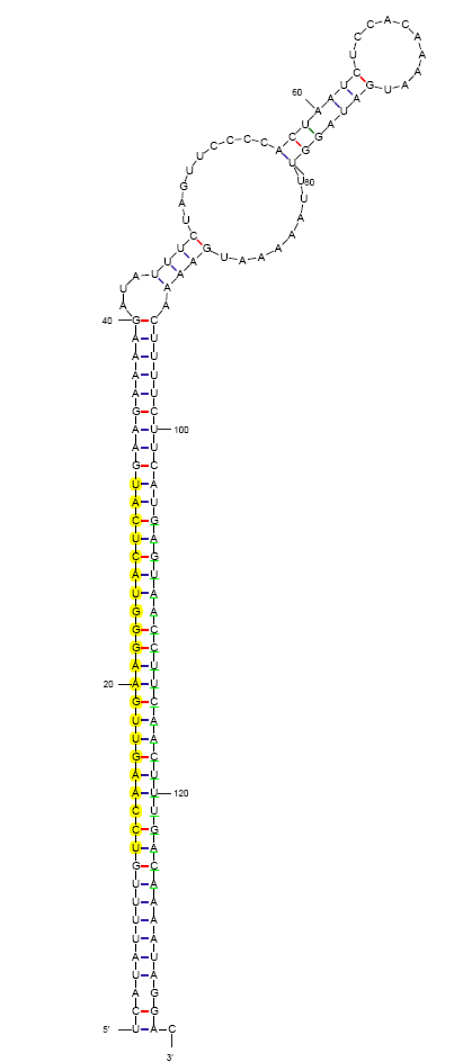
UCAUAUUUUGUCCAAGUUGAAGGGUACUCAUGAAGAAAAGAUAUUUCUAGUUCCCCACUAAUCUCCACAAAAUGAUAGGUUUAAAAAUGAAAACUUUUCUUCAUGAGUAACCUUCAACUUUGACAAAAUAGGAC

**novel230**

UAAUUCGUAUGAGAUAAGAGUUUGUCAUCUUCAUUGUGAAGCGUACAAGAAAGCUCGAUGCAUCUUGCAGCUGAGAAGAAUAUUGGUGGUCUCAGAAUGAAGAUGACAAACUCUAUCUCGUACGAAUU

**novel261**

GGCUUUAAGGAAGUUGUUGGAAUGGAAGGGUAGGAAAGAAGAAACUUCUCUUCUAUUUUCCAAAUUCAAGAGAGAAAAUUUCUUUCCUAUCCCUCCCAUUCCUUCAAUUUGCUAAAGUUUCU

**novel289**

CAACAGGCCAUCUGAAAAUCAGAUCUGACAAACCACCGCAAUUCGCCAUGGCAAGCCAGGAUAGGAAUGAGAUUUCACCAAACUCGCAGCACACCCUCCUCCUUCUCUCAAUAGACUUGUCUUCUCCCUCAAAUGGUAAAUAGGAUGAAAGAAUGGACAAAUCCGUUAAGAGAAAAAGGAGAGCGAGCCGCGAGUUUGGUCAAAUCUCACACCUACCCUUGCUAACCAUGGCGGAGUGCAGUGGUUUGUCAGAUCUGAUUUUCAGAUGGCCAGUUGG

**
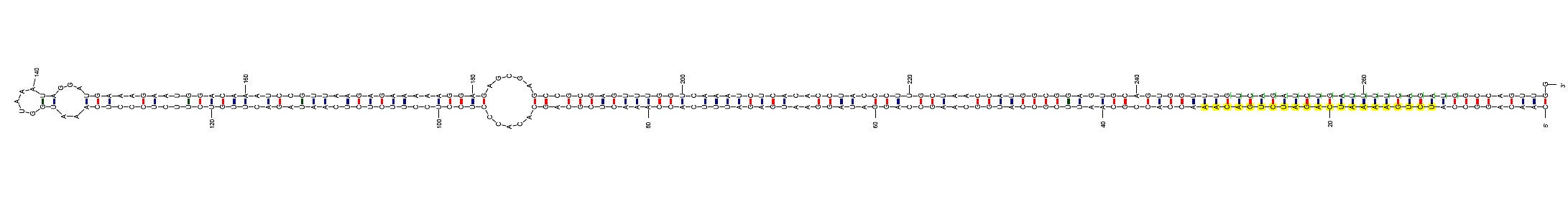
**

**novel292**


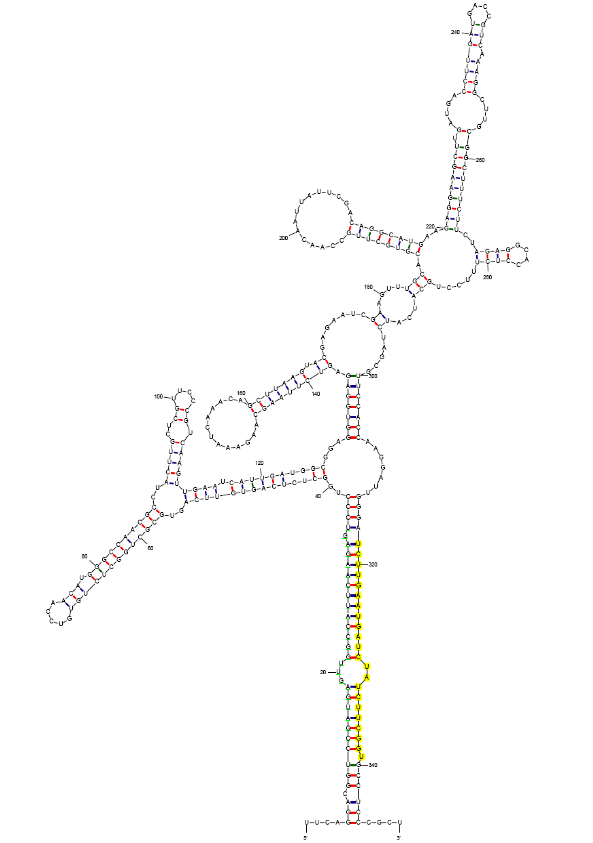
UUCAGGACGGUCCGAUGAGUUGGCCAUUCAAGAGUCCCUGGCUCUCAGUGUUCAGUGCGCUGGCUCUGUGUCCAACAUGGGCCAACGCCUACUUGCUCGUUCCCGUCAAGUUGAAUCAUUGAUGGCGGAGGUGGAGAGUCUUAAGCAAGAAAUCAAACAGCUUAAGUACGAGAAUCGAAGUUUGCACGUGCUUGCCAACAAUUAUUCGACAGGCAUGAAGAGGAAGCUUGAUGACCUUGAUGACCGUCAAAGGCUUGCGGCUUUCUUCUAGAGGCACCUCUUUCCUGCAUCAUCUAGCGUUCCACCAAGGAUUGGGAUCUUGAAUGAUCUAUCUUCGGUGCCUCCCGCU

**novel3**

GGAUGAAUAACAGUGCAAUUCUCCUUUGGCAGAAGUCAUUCUGGUGCAUGUACUCUUGAGUACUGUCGCUGGAUACAUAUAUAUGCUUUCUGCCAAAGGAGAAUUGCCCUGCAAUUCAAAA

**novel349**

AUCUUCAACGUGGUGUUUGGAUGGACGUGUUAAGAUCCUACAAAAUUUUCAUUUCUUUGAAAAUAUUUGUACAAGGUGAAUCACUCGGAGUACAAAAUCAGAGGAAAAUUUUUGGUACUUCUCAUACUUUUGAGGGGUUCACCUUGUUGUAAGAUCUUAAUAAGUUCAUCCAAACACCAUAUUGAGGAUG

**novel_mir_37**

**novel37**

UGCACCUCCAACAGUUGAAGCUGCCAGCAUGAUCUAAGCUAUCUUUGUAUACAGGAAAGGCUAUAGAUCAUGUGGUAGCUUCACCUGUUGAAUGAAGCAC

**novel370**

AGAGUAGAUAUGAGCCAAGGAUGACUUGCCACAAUCUCUAGUAUGUGAAUGCCCUUCAGUUCCACUUGGUAUGCUUUGACAAGUCAUCCUUGGUUCACCUUUGCUCUC

**
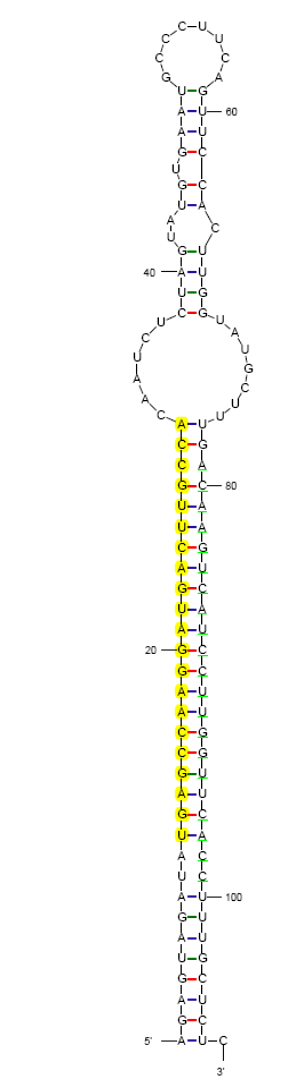
**

**novel371**

GAGUGCAAUGGAGCCAAGGAUGAAUUGCCGGCGCUAUAGAUAAUUAAGUUCAUAAAUGUGUAUACAUGCCACCAAAAUCCGUAUAUAUUUGCUUGCCGGCAAUUUGUUCUUGGCUAUGUUGGGUUCU

**novel39**

AUGAGCAUUGCAUGGGUAAUCAAUUUCAUCCGCUUAAUAAAUGCUUCUGCUAAAUCAUUUUUGAGUAUGAACAUGAGGAACAUGGUGUUCA

**novel43**

CUUAAGCUUUAACAACCUUUCAAAAACAGAGCUUUGGGAUGUUGGCGCGUUAAGCCUCUUGAAGACAGGGGUUCGGUCUUGACCACUAAUGCGAUUAAGCACUGAAGUUCUGGAGCUUGAA

**novel87**

UACACUUAAUACAUUUGUACCGACUCUCCAAUAGAGGUGGGUCCCACCUCUAUUGGAGAGGCGGUACAAAUGUGGUAAGGAUA
