# Supplementary material for: Identification and characterization of microRNAs from in vitro-grown pear shoots infected with Apple stem grooving virus in response to high temperature using small RNA sequencing
Source: BMC Genomics. 2015 Nov 16;16:945. doi: 10.1186/s12864-015-2126-8 (PMC4647338; doi:10.1186/s12864-015-2126-8)
Supplement: Additional file 7: Table S5. — The 109 candidate novel miRNAs detected in P. pyrifolia. (DOC 222 kb) [file 12864_2015_2126_MOESM7_ESM.doc]

***Table S5 The109 candidate novel miRNAs detected in P.pyrofolia***

| miRNA name | Length of  mature(nt) | Mature sequence( 5′→3′) | Counts of miRNAs | Precursor location | Length of precursor (nt) | Mature sequence locus (Arm ) | MFE  (kcal/mol) |
| --- | --- | --- | --- | --- | --- | --- | --- |
| novel124 | 21 | TGCCAAAGGAGAGTTGCCCTG | 63 | scaffold205.0:141218:141325:- | 108 | 3p | -43.20 |
|  | 21 | TGCCAAAGGAGAGTTGCCCTG | 63 | scaffold594.0:182727:182834:+ | 108 | 3p | -43.90 |
| novel125 | 21 | TGAAGGGAGAAATGGATGACA | 30 | scaffold21.0:1998:2194:- | 197 | 5p | -97.30 |
| novel147 | 23 | ATGTAGAAGTACACGACGTGTCA | 40 | scaffold246.0:642401:642670:+ | 270 | 5p | -60.80 |
| novel166 | 22 | GTAAGGATGCAAGTAGGGATCA | 39 | scaffold277.0:310417:310601:- | 185 | 3p | -45.80 |
| novel168 | 22 | TAAATCACTAGACTCGAGGCAT | 85 | scaffold278.0:159408:159668:+ | 261 | 5p | -133.40 |
| novel177 | 21 | TTACATTACAATCATCATTAG | 118 | scaffold290.0:481870:481968:+ | 99 | 5p | -23.20 |
| novel188 | 21 | CGGTGACAGAAGAGAGTGAGC | 42 | scaffold3.0:1985646:1985793:+ | 148 | 5p | -63.70 |
| novel19 | 21 | GGAGTGACCTGAGATCACATA | 825 | scaffold1049.0:16097:16179:- | 83 | 5p | -50.50 |
| novel190 | 23 | TAACACAGACTGATACACTAAAT | 45 | scaffold31.0:276763:276886:+ | 124 | 5p | -44.59 |
|  | 23 | TAACACAGACTGATACACTAAAT | 45 | scaffold873.0:143667:143790:+ | 124 | 5p | -43.69 |
|  | 23 | TAACACAGACTGATACACTAAAT | 45 | scaffold873.0:14371:14494:- | 124 | 5p | -43.69 |
| novel202 | 22 | TCTGACTTAAGAGAATGGAAGA | 31 | scaffold338.0:60544:60620:- | 77 | 3p | -19.80 |
| novel203 | 23 | TGTGGAATGGAAGGAGAATGGAT | 139 | scaffold34.0:1259907:1260059:+ | 153 | 3p | -45.50 |
|  | 23 | TGTGGAATGGAAGGAGAATGGAT | 139 | scaffold34.0:1294778:1294930:+ | 153 | 3p | -45.50 |
| novel228 | 23 | TGAGAATTGTTATTGGCACTCCA | 33 | scaffold4.0:564785:564928:- | 144 | 5p | -53.20 |
|  | 23 | TGAGAATTGTTATTGGCACTCCA | 25 | scaffold124.0:713453:713609:+ | 157 | 3p | -31.02 |
| novel233 | 22 | TTGTTGGAATGTTTAGGTGTAA | 34 | scaffold41.0:691160:691236:- | 77 | 3p | -25.20 |
| novel246 | 22 | TAGCCGAGGATGATTTGCCTGC | 36 | scaffold432.0:23508:23638:+ | 131 | 5p | -43.71 |
| novel247 | 23 | TAGGACATTCTAGAAGAATTTGA | 29 | scaffold432.0:144980:145237:+ | 258 | 3p | -122.30 |
| novel250 | 22 | GGCAGTTGTCCTTCGGCTAATC | 34 | scaffold44.2:437600:437739:- | 140 | 3p | -57.40 |
| novel26 | 23 | AATTGACGTTAAATTGTGAGGTA | 24 | scaffold112.0:249158:249438:- | 281 | 5p | -56.85 |
| novel266 | 23 | ACAATGGATCCACTAAAACAGCA | 327 | scaffold489.0:159566:159675:- | 110 | 3p | -59.00 |
|  | 23 | ACAATGGATCCACTAAAACAGCA | 327 | scaffold64.0:880045:880154:+ | 110 | 3p | -54.80 |
| novel27 | 23 | AGTTCGACTTATGTAGATGGCTT | 37 | scaffold1127.0:82618:82936:+ | 319 | 3p | -77.40 |
|  | 23 | AGTTCGACTTATGTAGATGGCTT | 37 | scaffold142.0:191863:192181:- | 319 | 3p | -77.40 |
| novel281 | 23 | AGTTCGAACTTCGTAGGTGGCTA | 36 | scaffold54.1:88708:88799:- | 92 | 3p | -28.50 |
| novel306 | 21 | TAAAAAGGGTTGTGTGGATAG | 60 | scaffold64.0:868621:868773:+ | 153 | 3p | -77.20 |
| novel308 | 23 | AACGAAAAGCCTACGGTACTGTT | 29 | scaffold64.0:1088707:1088920:+ | 214 | 3p | -49.90 |
| novel312 | 23 | TGAGTTGTTGGAATGTTTAGGCA | 26 | scaffold663.0:187352:187486:- | 135 | 5p | -50.50 |
| novel337 | 21 | GGTATACAAAGTCAACAGATC | 495 | scaffold80.0:102843:102960:- | 118 | 3p | -29.20 |
| novel338 | 22 | TTTGGATTGGAAAGATAAGGTT | 45 | scaffold80.0:872929:873062:- | 134 | 3p | -68.30 |
| novel348 | 22 | TTTTTGGATAGGAGGGGAGGTG | 35 | scaffold848.0:130625:130809:+ | 185 | 3p | -71.76 |
|  | 22 | TTTTTGGATAGGAGGGGAGGTG | 35 | scaffold848.0:68056:68240:- | 185 | 3p | -71.76 |
| novel363 | 21 | GTGACAGAAGAGAGTGAGCAC | 21 | scaffold94.0:516128:516232:+ | 105 | 5p | -52.80 |
|  | 21 | GTGACAGAAGAGAGTGAGCAC | 24 | scaffold3.0:1985648:1985754:+ | 107 | 5p | -56.50 |
| novel379 | 21 | AGGCGATGAAGAAGAAAACAT | 34 | scaffold101.0:744867:744996:+ | 130 | 3p | -23.00 |
| novel385 | 21 | AGCTGCCGACTCATTCACTCA | 11 | scaffold112.0:305682:305818:- | 137 | 5p | -44.80 |
|  | 21 | TGTGAATGAAGCGGGAGATAT | 48 | scaffold112.0:305682:305818:- | 137 | 3p | -44.80 |
| novel393 | 21 | CATGATTGGATTGGAATGGAG | 38 | scaffold12.0:1089501:1089786:- | 286 | 5p | -119.40 |
|  | 21 | CATGATTGGATTGGAATGGAG | 38 | scaffold12.0:1098053:1098339:- | 287 | 5p | -122.90 |
| novel395 | 21 | TTCACTTGAAGATCGTTCCTA | 48 | scaffold1216.0:84252:84457:+ | 206 | 5p | -67.10 |
|  | 21 | TTCACTTGAAGATCGTTCCTA | 48 | scaffold731.0:205272:205477:+ | 206 | 5p | -67.10 |
|  | 21 | TTCACTTGAAGATCGTTCCTA | 48 | scaffold731.0:81600:81805:- | 206 | 5p | -67.10 |
| novel412 | 23 | GTTCATTGTACATCGTGCGGTCA | 84 | scaffold137.0:397576:397821:+ | 246 | 3p | -47.90 |
| novel413 | 23 | AAGTTGGGTGTAGAAATAGGTTA | 39 | scaffold138.0:605384:605609:- | 226 | 3p | -43.20 |
|  | 23 | AAGTTGGGTGTAGAAATAGGTTA | 29 | scaffold323.0:221180:221358:- | 179 | 5p | -28.80 |
|  | 23 | AAGTTGGGTGTAGAAATAGGTTA | 39 | scaffold37.0:855449:855741:- | 293 | 3p | -60.20 |
| novel421 | 21 | CGGGAAGACTCACAGAGGCAT | 21 | scaffold150.0:108252:108351:- | 100 | 5p | -22.30 |
| novel423 | 21 | TCATTGATTTTCGAGAATTAC | 44 | scaffold156.0:132777:132938:+ | 162 | 3p | -43.90 |
| novel429 | 22 | TCAGTGATAATAGGAAGAGCAA | 23 | scaffold164.0:589916:590115:+ | 200 | 5p | -41.30 |
| novel438 | 22 | GTTCACGTGACTGAACTCGGCA | 116 | scaffold170.2:115946:116158:+ | 213 | 5p | -59.40 |
| novel439 | 23 | GCCTCGGAGTGCTTCGTTGGACA | 22 | scaffold171.0:315538:315755:- | 218 | 3p | -59.80 |
|  |  | GCCTCGGAGTGCTTCGTTGGACA | 22 | scaffold447.0:368432:368649:- | 218 | 3p | -59.80 |
| novel447 | 23 | TTAACCGTGACCGTACAAAATAA | 42 | scaffold18.0:433237:433381:- | 145 | 3p | -40.20 |
| novel457 | 22 | TGAATGATGGCCTATGACTCCC | 33 | scaffold197.0:539053:539298:- | 246 | 5p | -67.69 |
|  | 22 | TGAATGATGGCCTATGACTCCC | 33 | scaffold34.0:496779:497024:+ | 246 | 5p | -67.69 |
| novel476 | 23 | AAGTTGGATGTAGAAAGAGGTTA | 94 | scaffold233.0:152195:152413:+ | 219 | 3p | -38.12 |
|  | 23 | ATGTAGAAGTACACGACGTGTCA | 12 | scaffold246.0:642401:642670:+ | 270 | 5p | -60.80 |
| novel482 | 21 | GAAAAGAAACGGCGTACCTTT | 165 | scaffold251.0:411498:411709:- | 212 | 3p | -61.99 |
| novel498 | 21 | CAAAGCTGAAGACCATGGGTT | 41 | scaffold301.0:287566:287807:+ | 242 | 3p | -66.04 |
| novel504 | 23 | GTACGATGTACGATGAATGGACA | 49 | scaffold308.0:39536:39668:+ | 133 | 39 | -45.70 |
| novel509 | 20 | GGCAGTCTCCTTGGCTAATC | 67 | scaffold328.0:155365:155484:+ | 120 | 3p | -52.70 |
| novel516 | 23 | TGAAATTTGATCCAATGACTACA | 30 | scaffold342.0:378778:378874:+ | 97 | 5p | -35.93 |
| novel52 | 23 | TAGATTGTAATACTAAGTGGCTA | 50 | scaffold1291.0:52980:53266:+ | 287 | 5p | -56.36 |
|  | 23 | TAGATTGTAATACTAAGTGGCTA | 50 | scaffold262.0:313000:313286:+ | 287 | 5p | -52.50 |
| novel526 | 21 | AAATGATTGACAGTGGACAAA | 142 | scaffold378.0:87556:87720:- | 165 | 3p | -58.31 |
|  | 21 | AAATGATTGACAGTGGACAAA | 142 | scaffold378.0:88565:88728:- | 164 | 3p | -64.00 |
| novel531 | 23 | CGGGTCAACAGTTCGACTGGCAC | 40 | scaffold39.0:285883:285973:+ | 91 | 3p | -42.40 |
| novel533 | 23 | CAACTTAGGACTCGTTGGGAAGT | 26 | scaffold4.0:1838849:1839052:+ | 204 | 5p | -66.60 |
| novel543 | 23 | ATGCAATGACATGTAGAAGGACA | 24 | scaffold44.1:311402:311533:+ | 132 | 3p | -25.70 |
| novel560 | 21 | AAAATTTGATTCAACGGCTAA | 22 | scaffold533.0:145177:145280:+ | 104 | 5p | -38.54 |
| novel573 | 23 | TCTGTATCTTGCGATTGCGACCA | 25 | scaffold604.0:195074:195200:- | 127 | 3p | -45.70 |
| novel579 | 23 | CAAAATGATTGATGGTAGACAAA | 41 | scaffold63.0:363770:363967:+ | 198 | 3p | -62.90 |
| novel585 | 23 | CTTTTAAAAGCAGCATGTAGGTT | 32 | scaffold66.0:222014:222100:- | 87 | 3p | -27.70 |
| novel586 | 22 | TGAAATAAGGATCAAAATGACA | 24 | scaffold66.0:332854:332980:- | 127 | 5p | -33.00 |
| novel598 | 23 | ATTCATCGTGCATCGTGCGGTCA | 37 | scaffold718.0:213274:213563:+ | 290 | 3p | -75.30 |
| novel6 | 23 | AAATTAATGAAGATCAAAGGACA | 306 | scaffold1.0:3495733:3495907:- | 175 | 5p | -43.23 |
| novel601 | 21 | GCCAGGATTGGGTTCAGGTAC | 26 | scaffold76.0:66058:66153:+ | 96 | 5p | -72.10 |
| novel603 | 22 | TTGGAAGTTAGTCACGGCTTAA | 66 | scaffold779.0:96088:96313:+ | 226 | 5p | -47.20 |
| novel69 | 21 | GGAATGTTGTCTGGCTCGAGG | 1049 | scaffold144.0:774926:775122:- | 197 | 5p | -78.90 |
|  | 21 | GGAATGTTGTCTGGCTCGAGG | 1214 | scaffold200.0:628639:628809:- | 171 | 5p | -68.82 |
|  | 21 | GGAATGTTGTCTGGCTCGAGG | 1047 | scaffold275.0:393002:393190:+ | 189 | 5p | -72.20 |
|  | 21 | GGAATGTTGTCTGGCTCGAGG | 1214 | scaffold67.0:1032896:1033067:+ | 172 | 5p | -67.79 |
|  | 21 | GGAATGTTGTCTGGCTCGAGG | 1214 | scaffold75.0:598615:598785:- | 171 | 5p | -65.04 |
| novel8 | 22 | GAGCAGTCTCCTCTTGGCAAAC | 89 | scaffold1.0:3618540:3618679:- | 140 | 5p | -49.50 |
| novel83 | 22 | TTGCGTTCCACTGATTCTTTCG | 24 | scaffold16.0:214370:214501:+ | 132 | 3p | -59.10 |
| novel84 | 23 | AATTTGATCTAACGGCTAAAAAT | 27 | scaffold16.0:1278137:1278227:+ | 91 | 5p | -36.80 |
| novel99 | 21 | CCAAGGAGTGTGGGCCGTTGG | 23 | scaffold189.0:22699:22765:- | 67 | 5p | -59.20 |
| novel113 | 23 | GGAACGAGCTGTTCCACGGGACA | 52 | scaffold2.0:3084527:3084621:+ | 95 | 5p | -36.20 |
| novel116 | 22 | TTGTTCACCGTAGGCAGTGGTT | 52 | scaffold2.0:3777558:3777647:+ | 90 | 3p | -21.90 |
| novel123 | 21 | TGAAGATGTGTTTGCAACTGT | 59 | scaffold205.0:347556:347765:+ | 210 | 3p | -120.90 |
|  | 21 | TGAAGATGTGTTTGCAACTGT | 53 | scaffold205.0:347557:347766:- | 210 | 3p | -105.12 |
| novel126 | 22 | TCAACCTGACGGGAGCGAACAA | 2238 | scaffold21.0:922450:922540:- | 91 | 3p | -20.56 |
|  | 22 | TCAACCTGACGGGAGCGAACAA | 2238 | scaffold21.0:930378:930468:- | 91 | 3p | -20.56 |
|  | 22 | TCAACCTGACGGGAGCGAACAA | 2238 | scaffold254.0:12758:12848:- | 91 | 3p | -20.56 |
|  | 22 | TCAACCTGACGGGAGCGAACAA | 2242 | scaffold768.0:104282:104372:+ | 91 | 3p | -20.56 |
|  | 22 | TCAACCTGACGGGAGCGAACAA | 2238 | scaffold768.0:115512:115602:- | 91 | 3p | -20.56 |
| novel127 | 23 | AATTGACGTTAAACTGTGAGGTA | 523 | scaffold211.0:201972:202216:+ | 245 | 3p | -51.20 |
| novel134 | 21 | TCAAATGATCCGGGCCTTTCA | 57 | scaffold222.0:500979:501111:+ | 133 | 5p | -69.90 |
|  | 21 | TCAAATGATCCGGGCCTTTCA | 44 | scaffold8.0:1407300:1407433:+ | 134 | 5p | -51.30 |
| novel158 | 23 | GCATGGAGGCATGAGGATGGACA | 122 | scaffold268.0:218547:218739:- | 193 | 3p | -44.44 |
| novel17 | 21 | AATAAAGTTGATATTGGTGTG | 444 | scaffold103.0:807736:807904:- | 169 | 3p | -93.10 |
| novel172 | 22 | TTAAGTACAGTACGGAGTCATG | 101 | scaffold282.0:137092:137161:- | 70 | 5p | -21.10 |
|  | 22 | TTAAGTACAGTACGGAGTCATG | 54 | scaffold249.0:126506:126575:- | 70 | 5p | -24.00 |
| novel186 | 23 | GTGTAGAGTGTAGAGGGGTGCTA | 113 | scaffold299.0:354790:354870:- | 81 | 3p | -33.10 |
|  | 23 | GTGTAGAGTGTAGAGGGGTGCTA | 123 | scaffold447.0:179360:179440:- | 81 | 3p | -33.10 |
| novel189 | 22 | GCTCATGTCTCTTTCTGTCAGC | 64 | scaffold3.0:2868101:2868208:- | 108 | 3p | -50.50 |
| novel211 | 23 | AGTTCATGGACTAAATCGGCACT | 187 | scaffold351.0:13145:13253:- | 109 | 5p | -21.59 |
| novel218 | 22 | TGTTTGAGGAAATTGAGACGTT | 89 | scaffold367.0:389501:389757:- | 257 | 5p | -96.60 |
|  | 22 | TGTTTGAGGAAATTGAGACGTT | 89 | scaffold620.0:192125:192381:+ | 257 | 5p | -99.75 |
| novel22 | 22 | CGACAAGACGAAGAGAATGGTA | 85 | scaffold1086.0:87127:87318:+ | 192 | 5p | -95.30 |
|  | 22 | CGACAAGACGAAGAGAATGGTA | 46 | scaffold684.0:171920:172116:- | 197 | 5p | -92.50 |
| novel222 | 23 | AGGGACGACATTGATTGATTTTA | 91 | scaffold384.0:240454:240742:- | 289 | 5p | -66.06 |
| novel225 | 23 | GTGACTGTCCACATCAGATGTCA | 195 | scaffold39.0:876295:876501:- | 207 | 3p | -70.00 |
|  | 23 | GTGACTGTCCACATCAGATGTCA | 102 | scaffold140.0:539877:539981:- | 105 | 3p | -40.10 |
| novel226 | 22 | TATGGGATATTAGGGTGATGGA | 71 | scaffold390.0:276680:276962:- | 283 | 5p | -69.50 |
| novel253 | 22 | AGGCGTTGGCTCATGTTGGACA | 112 | scaffold457.0:134220:134412:- | 193 | 5p | -63.60 |
| novel256 | 21 | GGCTAACTCATCGGACCGTCC | 296 | scaffold469.0:10805:11055:- | 251 | 5p | -83.00 |
| novel257 | 22 | TACTGGCCTGGCATGAAAAGAG | 162 | scaffold473.0:207933:208258:+ | 326 | 3p | -94.80 |
|  | 22 | TACTGGCCTGGCATGAAAAGAG | 162 | scaffold473.0:296258:296583:- | 326 | 3p | -94.80 |
| novel260 | 21 | TTGGGGAAGACCACTCTCGCC | 60 | scaffold475.0:139477:139650:- | 174 | 3p | -48.90 |
|  | 21 | TTGGGGAAGACCACTCTCGCC | 60 | scaffold681.0:19671:19844:+ | 174 | 3p | -48.90 |
| novel265 | 22 | TTTAGGATGAACAGATGATGAA | 183 | scaffold487.0:112937:113020:+ | 84 | 3p | -22.00 |
| novel276 | 22 | CGATCTTTGTCGTTGACGGTGT | 1355 | scaffold52.0:99128:99488:- | 361 | 3p | -107.80 |
| novel28 | 21 | AGGGTGGTTACCAATGGGATG | 1401 | scaffold113.0:437650:437768:+ | 119 | 3p | -61.12 |
| novel280 | 21 | TGTTGAAATCAAGGCTTCTGC | 64 | scaffold535.0:290933:291079:+ | 147 | 5p | -36.80 |
| novel296 | 23 | AGAGAAGACGACGGAATATTCCA | 35 | scaffold608.0:110529:110604:+ | 76 | 5p | -26.30 |
| novel309 | 23 | TGCTCACTTCTCTTTCTGTCAGC | 99 | scaffold64.0:452413:452516:- | 104 | 3p | -56.00 |
| novel317 | 21 | CCGGATTTCGTGGTCAGGAGG | 203 | scaffold69.0:1033952:1034099:+ | 148 | 3p | -70.20 |
| novel319 | 22 | TTTGGTGGAACGCTAGATGACG | 109 | scaffold704.0:113496:113651:+ | 156 | 3p | -40.50 |
|  | 22 | TTTGGTGGAACGCTAGATGACG | 109 | scaffold704.0:129291:129446:+ | 156 | 3p | -40.50 |
| novel327 | 22 | AGTTACACTGACTGTTGAGGAA | 125 | scaffold74.0:82938:83033:+ | 96 | 5p | -19.86 |
| novel331 | 21 | TTTGGTTGGTAGTTTGATGTA | 58 | scaffold742.0:196581:196790:+ | 210 | 5p | -52.51 |
| novel336 | 21 | CAAAGGAAAGAACATGGAGAA | 42 | scaffold790.0:105801:106131:+ | 331 | 5p | -88.80 |
| novel343 | 23 | TTGGAATTGGAAGGAAATGCATA | 72 | scaffold815.0:156360:156527:- | 168 | 5p | -54.84 |
| novel351 | 23 | TGAAAACACAGCTCGGTACACCA | 37 | scaffold887.0:72068:72137:+ | 70 | 5p | -22.90 |
| novel352 | 22 | TCTTGAGAGGATGCACTGTTTT | 79 | scaffold888.0:134510:134639:+ | 130 | 5p | -32.70 |
| novel4 | 21 | TCACACTATGGAGCGATGGTC | 111 | scaffold1.0:57557:57709:- | 153 | 5p | -73.95 |
| novel60 | 22 | CAACTTTACTTGGATTGTTGAT | 434 | scaffold139.0:251700:251901:+ | 202 | 5p | -37.00 |
|  | 22 | CAACTTTACTTGGATTGTTGAT | 434 | scaffold173.0:381273:381399:+ | 127 | 5p | -21.80 |
|  | 22 | CAACTTTACTTGGATTGTTGAT | 434 | scaffold173.0:700494:700620:- | 127 | 5p | -21.80 |
| novel67 | 23 | AATAGGCGTAGATAGACCGTGGG | 957 | scaffold144.0:39341:39536:+ | 196 | 5p | -53.81 |
|  | 23 | AATAGGCGTAGATAGACCGTGGG | 957 | scaffold413.0:336064:336253:- | 190 | 5p | -55.79 |
| novel71 | 22 | TAGTGGAACGCTAGATGACGCA | 3918 | scaffold1456.0:34639:34810:- | 172 | 3p | -50.40 |
|  | 22 | TAGTGGAACGCTAGATGACGCA | 3918 | scaffold36.0:734774:734945:- | 172 | 3p | -50.40 |
| novel82 | 21 | TGGCCTTGGTGGAAGAGATCC | 27 | scaffold152.0:515755:515824:- | 70 | 5p | -21.60 |
|  | 21 | TGGCCTTGGTGGAAGAGATCC | 38 | scaffold152.0:515784:515969:- | 186 | 3p | -98.00 |
| novel90 | 21 | ACGGAGCCATGCGTTGAGCAC | 47 | scaffold166.0:229261:229531:+ | 271 | 3p | -72.96 |
| novel91 | 22 | CTGAACACTGAGAGCTAAGGAG | 487 | scaffold168.0:267454:267581:+ | 128 | 5p | -35.70 |
