# Supplementary material for: Identification and characterization of microRNAs from in vitro-grown pear shoots infected with Apple stem grooving virus in response to high temperature using small RNA sequencing
Source: BMC Genomics. 2015 Nov 16;16:945. doi: 10.1186/s12864-015-2126-8 (PMC4647338; doi:10.1186/s12864-015-2126-8)
Supplement: Additional file 8: Figure S2. — First nucleotide bias in novel miRNA candidates isolated from in vitro-grown pear shoots; (A) the 24 °C library, and (B) the 37 °C library. (DOC 66 kb) [file 12864_2015_2126_MOESM8_ESM.doc]

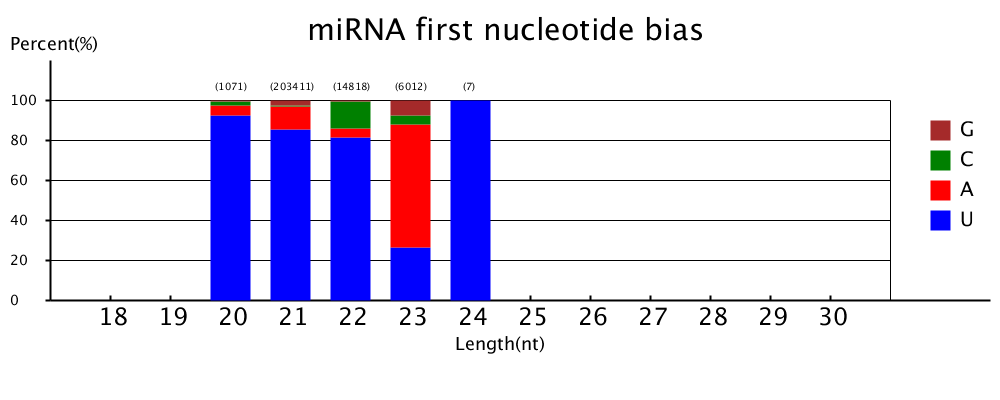

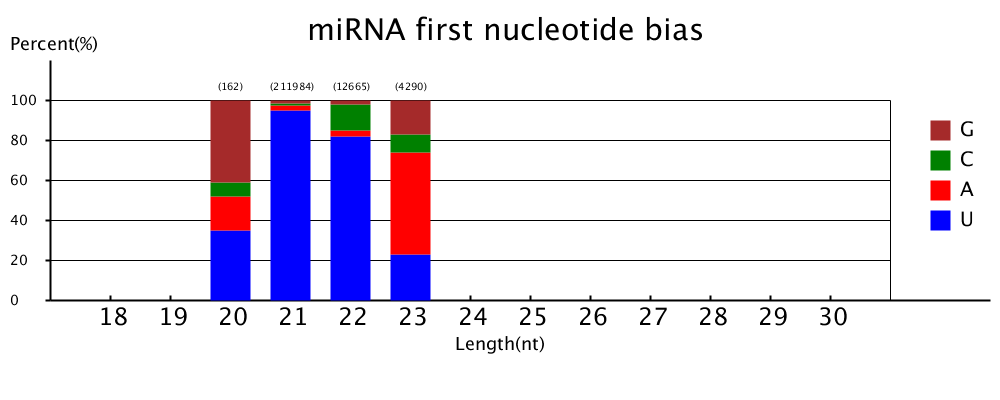

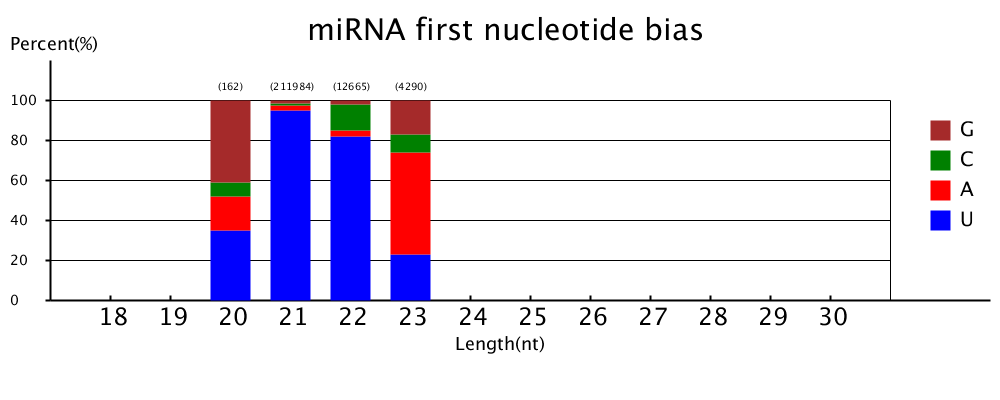

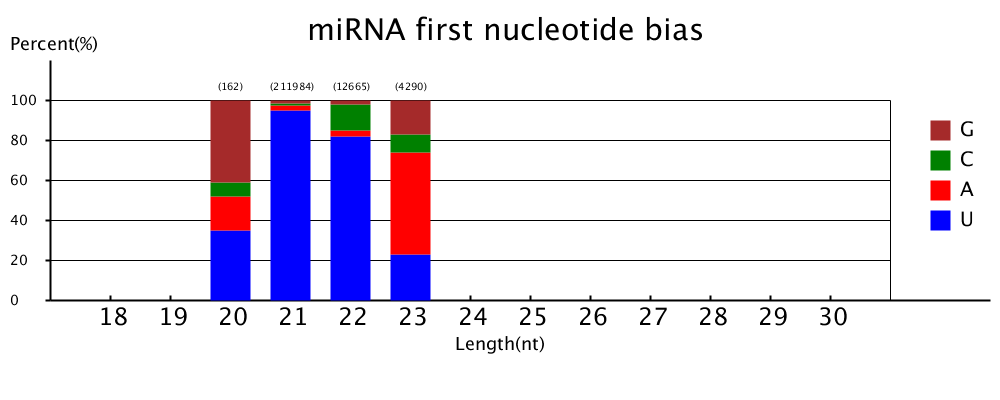
**Figure S2** First nucleotide bias in novel miRNA candidates isolated from *in vitro*-grown pear shoots; (A) the 24°C library, and (B) the 37°C library.

Percent (%)

Percent (%)

A

B
