# Supplementary material for: Identification and characterization of microRNAs from in vitro-grown pear shoots infected with Apple stem grooving virus in response to high temperature using small RNA sequencing
Source: BMC Genomics. 2015 Nov 16;16:945. doi: 10.1186/s12864-015-2126-8 (PMC4647338; doi:10.1186/s12864-015-2126-8)
Supplement: Additional file 10: Table S6. — Oligonucleotide primers used for qRT-PCR expression analysis of miRNAs, mRNAs, and ASGV cp genes isolated from in vitro-grown shoots of P. pyrifolia. (DOC 50 kb) [file 12864_2015_2126_MOESM10_ESM.doc]

**Table S6** Oligonucleotide primers used for qRT-PCR expression analysis of miRNAs, mRNAs, and ASGV genes isolated from *in vitro*-grown shoots of *P. pyrifolia*.

| **Name** | **Primers (5′-3′)** | **Size of primers (bp)** |
| --- | --- | --- |
| **Conserved miRNAs** | | |
| miR397a | TCATTGAGTGCAGCGTTGATG | 21 |
| miR477b | ACTCTCCCTCAAGGGCTTCGA | 22 |
| miR5519 | TGGCAGAGAGACTGGACTTGT | 21 |
| miR3627 | TTGTCGCAGGAGAGATGGTAC | 21 |
| **Novel miRNAs** | | |
| novel177 | GGGTTACATTACAATCATCATTAG | 24 |
| novel188 | CGGTGACAGAAGAGAGTGAGC | 21 |
| novel197 | CTCTTGACCGTTAGATTTGGCTT | 23 |
| novel241 | TGGTGCAGGTCGGGAACCGCT | 21 |
| novel262 | AGTGGAAGGGTAGGAAAGAAG | 21 |
| novel345 | TTGCATATCTCAGGAGCTGC | 20 |
| novel482 | GGAAAAGAAACGGCGTACC | 19 |
| novel566 | AGGTGAGACGTATCCCGAGGACA | 23 |
| Reverse primer |  | The kits provided |
|  | **Targets (mRNAs)** | |
| pbr002489-F | ATTTGGTGTTTCGTTCCTGTT | 21 |
| Pbr002489-R | TTCCAACCGCCATAGTTTCCA | 21 |
| pbr035962-F | GCCAGGGAAGACGATAATGT | 20 |
| pbr035962-R | GGGAATTGGACATTGTGTGA | 20 |
| pbr027204-F | GGTGGAGGAAGCGAAAGTAG | 20 |
| pbr027204-R | ATGATCCCTGAAATGAAGCC | 20 |
| pbr019211-F | GTCCAACAGAACCACACCAG | 20 |
| pbr019211-R | TCACAACGCCGGTAGAAAGTCA | 22 |
| pbr017710-F | GCAGCAGACATCGGAAATAG | 20 |
| pbr017710-R | TGTTTCCGCGTAGACCGTTG | 20 |
| pbr023226-F | CACTTGCTCTTTCACTGGGA | 20 |
| pbr023226-R | CAGGAGGAGCTTTCACACAA | 20 |
| pbr025376-F | AAAGAATCACCGCACTACCC | 20 |
| pbr025376-R | TCTTCAAGATTCACGCATCCA | 21 |
| pbr030437-F | TGGGCATTGAAAGTTATGGA | 20 |
| pbr030437-R | AATCAAGACACCGTTGACCA | 20 |
| pbr042600-F | TGGATTTAGTGTGCTATCGT | 20 |
| pbr042600-R | AGAGCTGAGGGATGATTCCGA | 21 |
| Actin-F | CCGGTTCATTACAATTTGACA | 21 |
| Actin-R | TGACAAGTCGATCCTCCAAA | 20 |
|  | **ASGV-*cp*** |  |
| ASGV-F | CTCCCAGGGCTGTGTTTCCTA | 21 |
| ASGV-R | CTCCATGTCATCCCAGTTGCT | 21 |
